# Inverse Vaccine Nanomimics: In situ Antigen-Specific Immune Tolerance Induction for Durable Cardiac Repair Post-Myocardial Infarction

**DOI:** 10.34133/research.1464

**Published:** 2026-10-21

**Authors:** Tian Yue, Jiali Yang, Qing Ma, Jian He, Ying Wang, Zhilu Yang, Jun Hou

**Affiliations:** ^1^ Department of Cardiology, The Third People’s Hospital of Chengdu/Affiliated Hospital of Southwest Jiaotong University, Chengdu Institute of Cardiovascular Disease, Chengdu, China.; ^2^ School of Ecology and Environment, Xizang University, Lhasa, China.; ^3^ The Tenth Affiliated Hospital, Southern Medical University (Dongguan People’s Hospital), Dongguan, China.

## Abstract

Alleviating the inflammatory response by inducing immune tolerance to cardiomyocyte lysate antigens has been shown to be a promising therapeutic approach for treating myocardial infarction (MI). However, immune tolerance induced by allogeneic antigens is constrained by insufficient antigen specificity, safety risks, and transient therapeutic efficacy. Herein, we present an injectable hydrogel capable of environment-responsive, long-acting delivery of inverse vaccine nanomimics (IVNMs) to overcome these challenges. These IVNMs efficiently capture antigens released by dying cardiomyocytes through the orthogonal reaction between surface maleimide groups and antigen thiols and promote mannose-facilitated uptake by CD206-expressing immune cells, including dendritic cells (DCs). The antigens captured by IVNMs, together with the sustained release of rapamycin from IVNMs, promote the differentiation of DCs into tolerogenic DCs, leading to a persistent increase in regulatory T cells and the establishment of immune tolerance memory. In post-MI rat models, myocardial inflammation was markedly and persistently alleviated, accompanied by enhanced neovascularization and reduced fibrosis, contributing to a sustained cardioprotective effect.

## Introduction

Myocardial infarction (MI) remains a leading cause of cardiovascular morbidity and mortality, imposing a substantial burden on healthcare systems worldwide [1]. Following MI, cardiomyocytes (CMs) undergo irreversible injury within 20 to 30 min of ischemia and hypoxia [2]. These necrotic CMs release cellular antigens known as cardiac lysate antigens (CLAs), which are captured and processed by dendritic cells (DCs), thereby initiating immune activation and amplifying the inflammatory response [3]. An excessive or prolonged inflammatory reaction can trigger pathological fibrosis, leading to further deterioration of cardiac function and increased risk of adverse outcomes [4–6]. To mitigate post-MI inflammation, various therapeutic agents including anti-inflammatory medications [7], monoclonal antibodies targeting proinflammatory cytokines [8,9], active constituents of traditional Chinese medicine [9–11], stem-cell or exosome therapy [12–14], nucleic acids, and bioactive ions [15–17] have been investigated for their cardioprotective effects against ischemic injury. Furthermore, clinical studies have shown that vaccination following MI is associated with improved clinical outcomes, suggesting that short-term post-MI interventions and the remodeling of immune response patterns are beneficial for recovery [18–20]. While these interventions demonstrate promising short-term efficacy, they lack the capacity to induce immunological memory and tend to cause systemic immunosuppression [21,22], thus failing to provide safe and sustained anti-inflammatory protection. As a result, they cannot prevent the recurrent inflammatory responses triggered by CLAs released from necrotic myocardial cells [23,24]. Hence, the development of a more effective strategy capable of suppressing inflammatory reactivation is essential for improving long-term myocardial recovery after MI.

After MI, DCs, T cells, including regulatory T cells (Tregs), and macrophages rapidly infiltrate the injured myocardial tissue via the endocardium, which functions as a “hidden channel” [25]. Regulating DCs and Tregs to establish immune tolerance renders the body unresponsive to specific antigens, a process with notable physiological implications for limiting excessive inflammatory responses and restoring immune homeostasis [26–28]. This breakthrough was awarded the Nobel Prize in 2025. Compared to conventional anti-inflammatory therapies, the strategy of modulating inflammation through induced immune tolerance offers several advantages: (a) high specificity, targeting only disease-relevant antigens [29]; (b) long-lasting effects-preventing recurrent inflammation [30,31]; (c) selective immunomodulation-preserving normal immune defenses [32,33]; (d) reduced reliance on high-dose or continuous drug administration-enhancing safety and minimizing adverse effects [33]; and (e) promotion of sustained immune homeostasis [31]. These attributes have shifted postinfarction inflammatory intervention from broad immunosuppression toward precise, mechanism-based prevention of pathological inflammation at its origin. Once immune tolerance to cardiac-specific antigens is established, the immune system can maintain a hypo-responsive state over the long term without ongoing treatment, while retaining protective immunity against pathogens and tumors [22,34]. Consequently, inducing tolerance to cardiac-specific antigens has emerged as a promising therapeutic approach for MI. Studies have demonstrated progress in controlling postinfarction inflammation through adoptive transfer of in vitro-generated tolerogenic DCs (tDCs) or in vivo delivery of antigen-targeted formulations combined with agents that modulate DC phenotype [35–37]. However, the in vitro generation and antigen loading of tDCs are technically complex, and tolerance induced by allogeneic antigens often suffers from limited antigen specificity, potential safety concerns, and transient efficacy, thereby constraining long-term outcomes [36]. Thus, identifying pathogenic antigen types and origins, enabling efficient antigen delivery, and ensuring sustained low-dose antigen exposure remain critical challenges in achieving durable immune tolerance.

Here, we report an injectable inverse vaccine platform that induces durable, antigen-specific immune tolerance in situ for MI therapy [38]. This system integrates environmentally responsive inverse vaccine nanomimics (IVNMs) with a degradable hydrogel (Gel) to achieve 3 coordinated functions: in situ capture of autologous CM-derived antigens released after ischemic injury, mannose-facilitated uptake of antigen-loaded IVNMs by CD206-expressing immune cells, and sustained immunomodulation (rapamycin [Rapa]) to promote tolerogenic DC differentiation. By eliminating the need for predefined antigen identification and ex vivo antigen loading, this strategy overcomes key limitations of existing tolerance-inducing approaches [39–41]. We demonstrate that a single local administration establishes long-lasting immune tolerance memory, suppresses inflammatory reactivation upon antigen restimulation, and enables sustained cardiac repair in vivo. These findings establish an engineering paradigm for reprogramming postinfarction immunity through in situ tolerance induction, offering a translationally relevant strategy for durable immunomodulation in cardiovascular disease (Fig. 1).

**Fig. 1.**
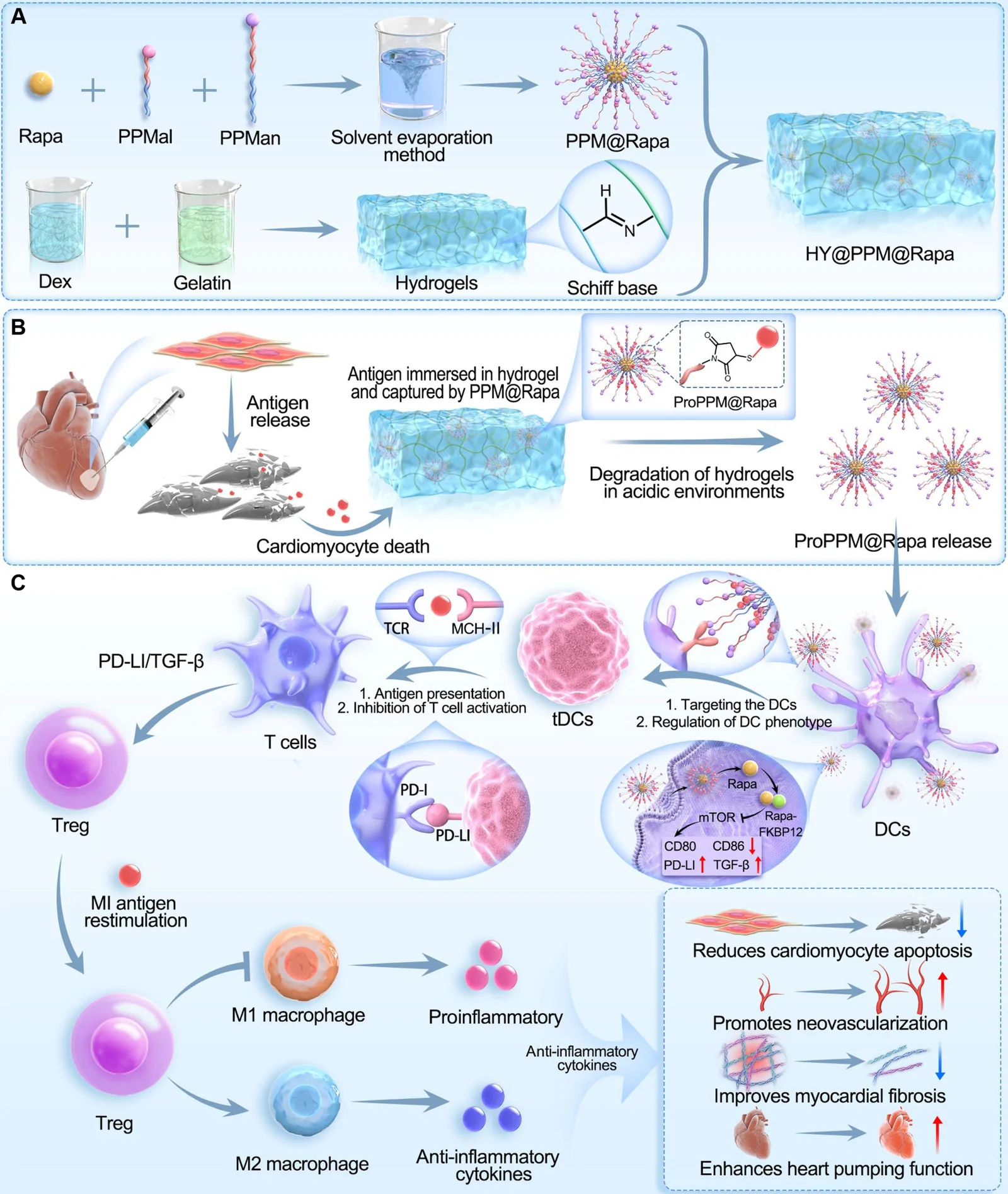
The fabrication of Gel@IVNMs, in vivo application and immunomodulatory mechanism for myocardial infarction (MI) therapy. (A) The stepwise fabrication of the PPM@rapamycin (Rapa) inverse vaccine nanomimics (IVNMs), Gel, and Gel@IVNMs. First, PPMal, PPMan, and Rapa are assembled into the IVNMs via the solvent evaporation method. Concurrently, oDex and gelatin are cross-linked to form a hydrogel network, where IVNMs are encapsulated. (B) The hydrogel is injected into the infarcted myocardium. As the Gel swells, cardiac lysate antigens (CLAs) released by necrotic cardiomyocytes diffuse into it and are captured by Mal on the surface of IVNMs to form antigen-loaded IVNMs (AIVNMs). Lactic acid accumulates at the infarcted site due to the activation of inflammation, followed by a decrease in pH, leading to a mild acidic microenvironment. In response to the mildly acidic pH, the Gel gradually degrades and releases AIVNMs. (C) Released AIVNMs can be taken up by CD206-expressing immune cells, including dendritic cells (DCs), and subsequently modulate DC phenotype by suppressing the mechanistic target of rapamycin (mTOR) pathway (via Rapa-mediated inhibition of mTOR–Rictor–FKBP12 signaling), reducing CD80 and CD86 expression, and increasing programmed death ligand 1 (PD-L1) expression and transforming growth factor-β (TGF-β) secretion. These DCs then present antigens and suppress T cell activation through PD-L1 signaling and TGF-β secretion, fostering regulatory T cell (Treg) expansion. Tregs further modulate macrophage polarization, shifting proinflammatory M1 macrophages toward anti-inflammatory M2 macrophages, thereby balancing proinflammatory and anti-inflammatory cytokine secretion. The established immune tolerance memory can rapidly respond and specifically inhibit inflammatory activation upon restimulation with CLAs. This cascade reduces cardiomyocyte apoptosis, promotes neovascularization, ameliorates myocardial fibrosis, and ultimately enhances heart pumping function, contributing to improved cardiac repair after MI.

## Results and Discussion

### Fabrication and characterization of IVNMs

In this study, polyethylene glycol (PEG) and polycaprolactone (PCL) were linked via ester bonds, with Mal and Man grafted onto the PEG termini, respectively, to form a micellar polymer. In an aqueous environment, this polymer self-assembled to encapsulate Rapa, resulting in the formation of IVNMs in the form of nanoparticles (Fig. 2A). Fourier transform infrared spectroscopy (FT-IR) results indicated that the infrared spectrum of COOH-PEG-COOH showed a stretching vibration of the C-O-C peak at 1,106 cm^−1^, while a characteristic carbonyl peak appeared at 1,732 cm^−1^. The absorption peaks at 2,860 and 2,943 cm^−1^ confirmed the successful polymerization of PCL. The infrared spectrum of PP-COOH showed both characteristic peaks of PCL and the C-O-C absorption peak of PEG, 1,106 cm^−1^, indicating the successful polymerization of PEG-PCL (PP). The infrared spectrum of PPMan showed no obvious difference in characteristic peaks compared with PP-COOH, but the stretching vibration peak of the amide bond at 1,540 cm^−1^ was slightly stronger than that of PP-COOH. Similarly, the infrared spectrum of PPMal also showed no obvious difference in characteristic peaks from PP-COOH, with the stretching vibration peak of the amide bond at 1,540 cm^−1^ being slightly stronger than that of PP-COOH (Fig. 2B). The proton nuclear magnetic resonance (^1^H NMR) results also confirmed the successful synthesis of the micellar polymer. The characteristic proton peaks of the benzene ring appeared at *δ* = 5.06 ppm (a+b+c), while the proton peaks at *δ* = 4.08 ppm (i), *δ* = 2.33 ppm (e), *δ* = 1.66 ppm (f+g), and *δ* = 1.40 ppm (g) correspond to the methylene protons in the PCL backbone, indicating the successful polymerization of PCL (Fig. S1A). The chemical shift at *δ* = 2.64 ppm (c+d) represents the characteristic proton peaks of the 2 methylene groups on the carboxyl group, confirming the successful grafting of carboxyl groups onto PEG2000 and PEG4000. The presence of characteristic benzene ring proton peaks at *δ* = 7.37 ppm (a+b+c) and the methylene peak of the PEG backbone at *δ* = 3.66 ppm (j) demonstrates the successful grafting of COOH-PEG-PCL. Additionally, the characteristic proton peak of the Mal ring at *δ* = 6.73 ppm (k) (Fig. S1A) verifies the successful modification of the Mal group, while the characteristic proton peak of the Man ring within the range of *δ* = 3.70 to 3.78 ppm (k) confirms the successful modification of the Man group (Fig. S1B).

**Fig. 2.**
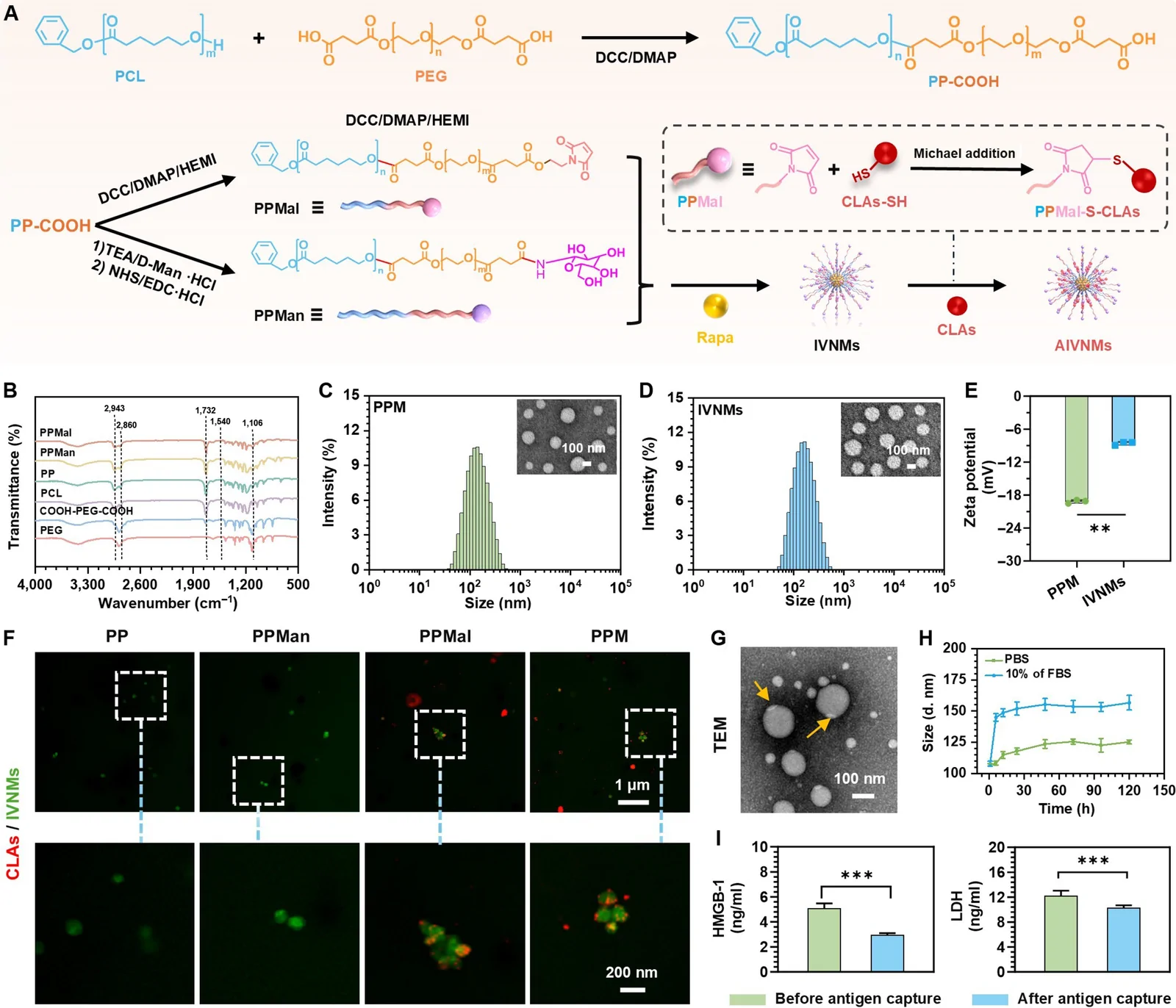
Fabrication and characterization of inverse vaccine nanomimics (IVNMs). (A) Schematic diagram of the synthesis route of nanomicelle polymers and their self-assembly into IVNMs and the antigen-capture reaction of Mal via Michael addition. (B) Fourier transform infrared spectroscopy spectra of nanomicelle polymers and intermediate products. (C and D) IVNM size distribution and transmission electron microscopy (TEM) images. (E) Surface potential of IVNMs before and after Rapa loading. (F) Fluorescence localization of IVNMs (green) and proteins (red) after coincubation with PP, PPMan, PPMal, and mixture of PPMan and PPMal (PPM) with antigens. (G) TEM image of PPM after antigen adsorption. (H) Stability of PPM in medium with or without FBS. (I) Enzyme-linked immunosorbent assay quantification of high-mobility group box 1 (HMGB1) and lactate dehydrogenase (LDH) before and after antigen adsorption by PPM. Data are presented as means ± SD, with individual points representing biological replicates. Statistical significance is indicated as ns, not significant; **P < 0.01; ***P < 0.001.

Changes in the ratio of Man and Mal groups can affect the antigen-capture capacity and mannose-facilitated cellular uptake of IVNMs. Therefore, balancing the ratio of Man to Mal is crucial. After the antigen was captured, the particle sizes of mixture of PPMan and PPMal (PPM), PPM (3:1), and PPM (2:1) all increased significantly with obvious differences. Meanwhile, the potential changes of PPM and PPM (3:1) also showed significant differences (Fig. S2A and B). As the content of PPMal decreased, the amount of captured protein also decreased. For micelles composed entirely of PPMal, the protein adsorption capacity of PPM reached 122.04 ± 16.67 μg/mg; when the ratio of PPMal to PPMan was 1:1, the adsorbed protein content decreased to 80.40 ± 5.91 μg/mg. These results indicated that PPM indeed could capture antigens, and the amount of captured protein decreased as the number of Mal groups was reduced (Fig. S2C). Considering the need to retain mannose-facilitated cellular uptake, PPM (3:1) was selected for subsequent experiments. When the Rapa release reached equilibrium, 64 ± 0.8% of Rapa was released from the IVNMs (Fig. S2D). Dynamic light scattering showed a normally distributed particle size, whereas transmission electron microscopy (TEM) showed uniform particles with an intact spherical morphology (Fig. 2C and D). When the mass ratio of Rapa to PPM was 1:5, the drug-loading efficiency of IVNMs was 18.07 ± 0.07% (Table S1). The surfaces of PPM and IVNMs carried negative charges (Fig. 2E).

Ideally, CLAs are adsorbed onto the surface of IVNMs and thus captured by IVNMs, which is corroborated by the results of this study. To visually observe the spatial distribution of CLAs captured by PPM, we performed fluorescent labeling of CLAs (Red) and PPM (Green). The results demonstrated that both PPMal and PPM with Mal groups exhibited obvious capture of CLAs on their surfaces, whereas the PP group and PPMan group without Mal groups failed to capture CLAs (Fig. 2F). This is consistent with the TEM results, which showed that CLAs were distributed on the surface of IVNMs (Fig. 2G). Furthermore, IVNMs maintained stable particle sizes after antigen capture in both phosphate-buffered saline (PBS) and medium containing 10% fetal bovine serum (FBS) (Fig. 2H). We used enzyme-linked immunosorbent assay (ELISA) to evaluate the concentration changes of key proteins, namely high-mobility group box 1 (HMGB1) and lactate dehydrogenase (LDH), in the environment before and after protein was captured by IVNMs. The results showed that the concentrations of HMGB1 and LDH in the conditioned medium were 5.12 ± 0.37 ng/ml and 12.34 ± 0.85 ng/ml, respectively, with differences of 2.40 ng/ml and 2.33 ng/ml compared with those after antigen adsorption. These findings indicate that PPM can capture proteins released from cells (Fig. 2I).

### Design, preparation, and characterization of Gel@IVNMs

We designed an injectable, pH-responsive hydrogel using Schiff-base cross-linking. Therefore, dextran was oxidized using sodium periodate to generate aldehyde-rich oDex, which subsequently reacted with amino groups in gelatin through Schiff-base formation to yield the Gel (Fig. 3A). FT-IR analysis confirmed the oxidation and cross-linking. oDex exhibited a characteristic aldehyde stretching band at 1,735 cm^−1^ (Fig. 3B). In contrast, the infrared spectrum of the cross-linked Gel displayed a C=N absorption at 1,640 cm^−1^ and a new Schiff base peak at 1,056 cm^−1^, while the aldehyde peak at 1,735 cm^−1^ disappeared, confirming Schiff base-mediated cross-linking.

**Fig. 3.**
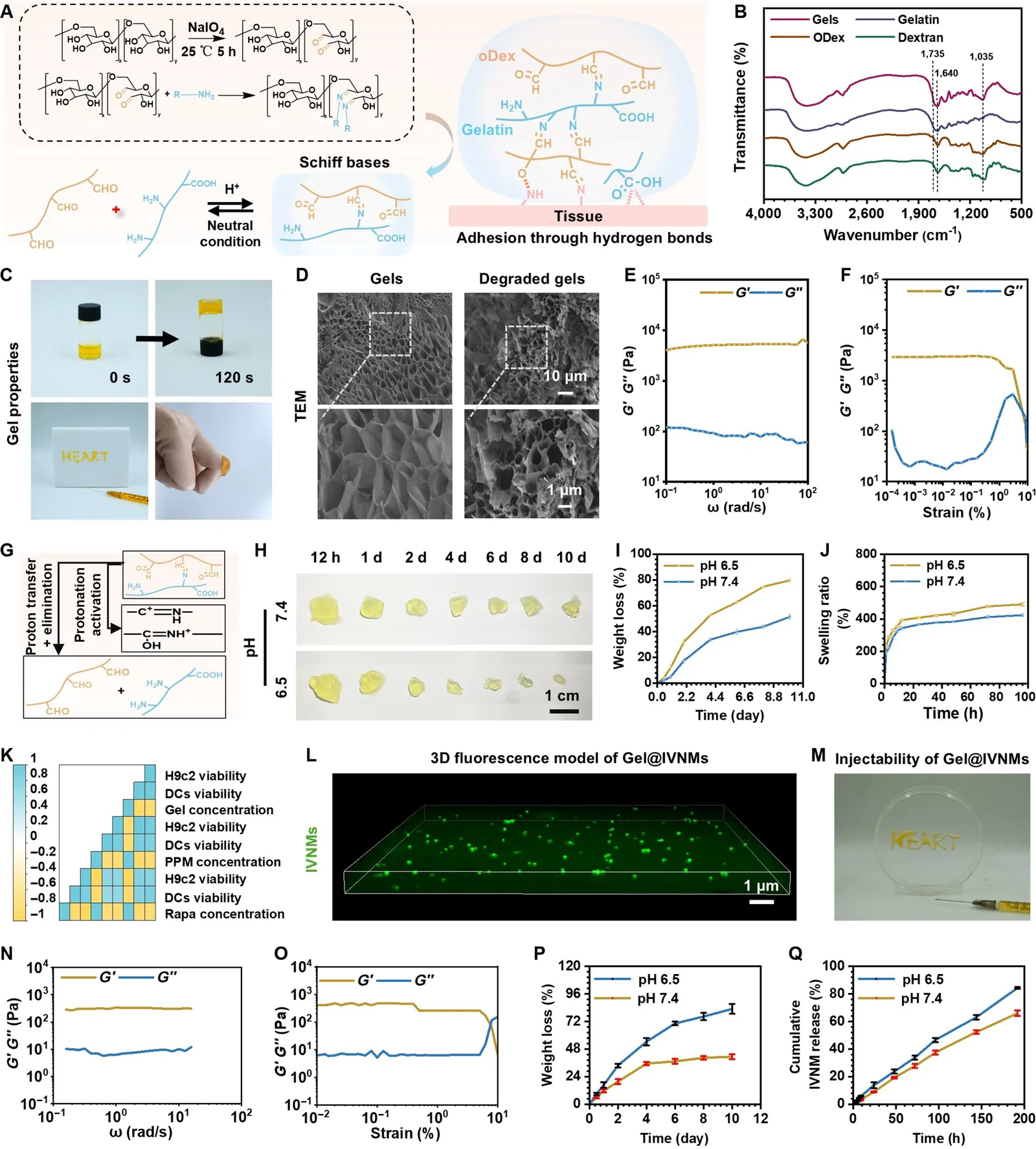
Preparation of injectable Gel@IVNMs. (A) Schematic diagram of precursor synthesis and the mechanism of Gel formation. (B) Fourier transform infrared spectroscopy of Gel. (C) Gelation time, injectability, and tissue adhesion properties of the Gel. (D) Scanning electron microscopy micrographs of Gel before and after network collapse. (E and F) Rheological properties of the Gel. (G) Mechanism of Schiff base cleavage under acidic conditions. (H) Residual amount of Gel after degradation over time at pH 6.5 and pH 7.4. (I) Degradation curves of Gel at pH 6.5 and pH 7.4. (J) Water absorption and swelling curves of Gel at pH 6.5 and pH 7.4. (K) Correlation analysis of biosafety and cytotoxicity of inverse vaccine nanomimics (IVNMs), Gel, and rapamycin (Rapa). (L) 3D diagram of IVNM distribution in Gel. (M) Injectability of Gel@IVNMs. (N and O) Rheological properties of Gel@IVNMs. (P) Degradation curves of Gel@IVNMs at pH 6.5 and pH 7.4. (Q) IVNMs release curves at pH 6.5 and pH 7.4. Data are presented as means ± SD, with individual points representing biological replicates.

After mixing the Gel precursor liquids, gelation occurred within 120 ± 10 s. The formed Gel could be smoothly injected through a 26G needle. In addition, the Gel adhered firmly and maintained adhesion after joint bending while applied to the skin surface (Fig. 3C). To evaluate internal microstructure, the gel was freeze-dried, fractured, and examined by scanning electron microscopy (SEM). As shown in Fig. 3D, the Gel presented a loose and porous 3-dimensional network favorable for drug loading and sustained release [42]. After 2 d of degradation, SEM showed that the network had loosened and partially collapsed, which facilitated release. We next evaluated the mechanical properties of Gel. The rheological analysis demonstrated solid-like behavior of the Gel, with storage modulus (*G*′) consistently higher than loss modulus (*G*″) across 0.1 to 100 rad/s, indicating a dominant elastic response and a stable network [43] (Fig. 3E). Specifically, at 1 Hz, changes in the dynamic modulus under shear strain were minimal until a critical strain of 6.7% (shear force 267 Pa), at which *G*′ intersected *G*″ and rapidly declined, indicating network disruption and an advantageous property enabling injectability [44] (Fig. 3F).

In our research design, the Schiff base bonds undergo protonation and break in a mildly acidic environment (pH = 6.5) (Fig. 3G). Since lactic acid accumulation in inflamed tissues reduces local pH, degradation profiles were compared between pH 7.4 and 6.5 [45]. It was shown that the Gel degraded significantly faster at pH 6.5 (Fig. 3H to J), enabling pH-responsive release advantageous in the MI microenvironment. Swelling behavior further supported this responsiveness: The swelling ratio increased over time and reached a plateau, with higher equilibrium swelling at pH 6.5 than pH 7.4, promoting antigen uptake.

Further, cytocompatibility and hemocompatibility assessments confirmed that IVNMs and Gel were biocompatible (Fig. S3A to D). Rapa showed limited cytotoxicity below 10 μg/ml, with half-maximal inhibitory concentration values of 23.99 μg/ml for H9c2 cells and 12.88 μg/ml for DC2.4 cells (Fig. S3E and F). Based on this analysis, 25 μg/ml was selected as a safe and effective IVNM concentration, and Gel@IVNMs was prepared accordingly. Three-dimensional fluorescence imaging revealed that IVNMs were uniformly distributed throughout the Gel (Fig. 3L). The Gel@IVNMs remained injectable through a 26G needle (Fig. 3M) without significant changes in mechanical properties, which were comparable to Gel alone (Fig. 3N and O). Meanwhile, the degradation profiles of Gel@IVNMs at pH 6.5 and 7.4 closely matched those of Gel (Fig. 3P), whereas the release of IVNMs was significantly accelerated under slightly acidic conditions (Fig. 3Q). In summary, Gel@IVNMs exhibits desirable injectability, mechanical stability, and pH-responsive degradation, enabling controlled release of IVNMs in the inflammatory MI microenvironment.

### CLAs promote DC maturation, while surface mannose modification enhances the uptake of IVNMs by DCs in vitro

To characterize DC phenotypes in vitro, we generated bone marrow-derived DCs from mice. As shown in Fig. 4A, DCs were obtained by isolating mouse bone marrow cells and culturing them with granulocyte-macrophage colony-stimulating factor and interleukin-4 (IL-4) [46]. Flow cytometry (FC) analysis confirmed successful DC differentiation, with CD11c^+^ cells accounting for 83.84% ± 3.11% of the population (Fig. S4A) [47]. When stimulated with CLAs, DCs exhibited robust activation, as indicated by a marked increase in CD86 and CD80 expression, with the CD86^+^/CD80^+^ ratio reaching 43.87% ± 2.44%, which was comparable to that in the lipopolysaccharide positive control (46.26% ± 4.09%). In contrast, DCs treated with Gel@PPM maintained a CD86^+^/CD80^+^ ratio of 25.37% ± 7.94%, which did not differ significantly from that in the control group (30.61% ± 5.72%), suggesting that Gel@PPM had a limited effect on DC activation (Fig. S4B). The extent to which DCs take up IVNMs can influence subsequent phenotypic modulation. Therefore, efficient cellular uptake is important for this strategy [30]. Furthermore, we sought to determine whether antigen loading reduced the uptake of IVNMs by DCs because of steric hindrance from surface-bound antigens. Thus, to investigate the effects of mannose functionalization and antigen loading on the uptake of IVNMs by DCs, Nile Red-labeled IVNMs were coincubated with DCs in this study. Fluorescent imaging revealed that free Nile Red produced weaker intracellular signals than PP@Nile micelles, with the positive signal increasing from 22.67% ± 1.67% to 29.33% ± 1.67%. This indicates that the micellar PP structure enhances cellular uptake of hydrophobic cargo. Additionally, mannose incorporation further increased the uptake of IVNMs by DCs, with PPM@Nile showing an additional increase of 10.67% ± 0.54% compared with PP@Nile. Uptake of APPM@Nile and PPM@Nile was comparable, indicating that antigen loading did not impair the uptake of IVNMs by DCs (Fig. 4B to D). Because CD206 expression is not restricted to DCs [30,34], these findings support enhanced uptake under the tested in vitro conditions rather than DC-specific targeting.

**Fig. 4.**
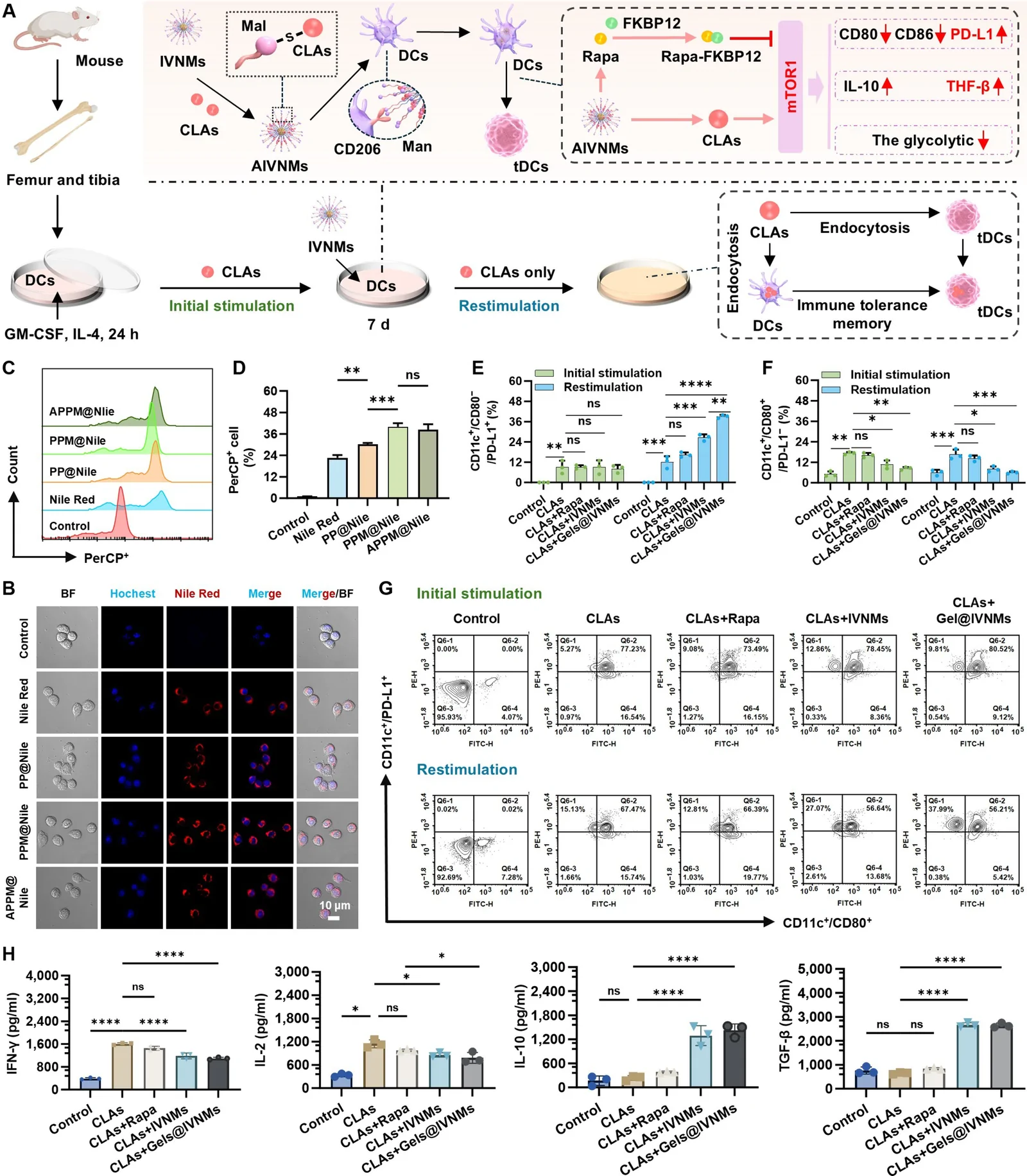
The effect of mannose functionalization on the uptake of inverse vaccine nanomimics (IVNMs) by dendritic cells (DCs) in vitro, together with DC phenotypes and cytokine secretion after stimulation with cardiac lysate antigens (CLAs). (A) DC isolation and experimental workflow. The process by which IVNMs capture antigens and are internalized by DCs. After antigen-loaded IVNMs (AIVNMs) are internalized by DCs, the antigens activate mechanistic target of rapamycin (mTOR) signaling, whereas Rapa inhibits mTOR activation, which leads to decreased expression of molecules such as CD80 and CD86, and increased expression of programmed death ligand 1 (PD-L1). Additionally, the secretion of anti-inflammatory factors (such as interleukin-10 [IL-10] and transforming growth factor-β [TGF-β]) is enhanced. (B) Flow cytometry results for the uptake of IVNMs by DCs. (C) Quantification of the uptake of IVNMs by DCs. (D) Fluorescence images of the uptake of IVNMs by DCs, with red fluorescence indicating IVNMs and blue fluorescence indicating cell nuclei. (E and F) Quantification of DC phenotypes by flow cytometry. (G) Representative flow cytometry plots of DC phenotypes. (H) Enzyme-linked immunosorbent assay detection results of interferon-γ (IFN-γ), IL-2, IL-10, and TGF-β expression levels in DCs after CLA stimulation and drug administration. Data are presented as means ± SD, with individual points representing biological replicates. Statistical significance is indicated as ns, not significant; *P < 0.05; **P < 0.01; ***P < 0.001; ****P < 0.0001.

### Gel@IVNMs modulates DC phenotype and supports CLAs-specific immune tolerance

During immune tolerance induction, DCs play a central role in antigen presentation and T cell phenotype regulation [30,42]. Therefore, how DCs change after phagocytosing CLAs is vital to this strategy, as it directly influences the process of immune tolerance induction and the subsequent activation status of inflammation. In this strategy, following antigen capture by IVNMs, DCs internalize antigen-loaded IVNMs through a process consistent with mannose-facilitated, CD206-associated uptake, after which intracellularly released CLAs and Rapa promote a CLAs-responsive tolerogenic phenotype. We therefore examined the phenotypic changes following stimulation. After the initial 24-h stimulation with CLAs, a significant increase in CD11c^+^/CD80^−^/PD-L1^+^ was observed, increasing from an undetectable level in controls to 9.04% ± 3.87%. However, inflammatory activation did not appear to affect the proportion of tDCs. Rapa, IVNMs, and Gel@IVNMs showed little effect on DC phenotype, suggesting that phenotypic tolerance had not yet occurred after initial phagocytosis of CLAs and that CLAs alone modestly induced PD-L1 expression (Fig. 4E to G, initial stimulation). In contrast, stimulation with CLAs significantly promoted DC maturation and inflammatory activation. Accordingly, CLA stimulation markedly increased CD11c^+^/CD80^+^/PD-L1^−^ activated DCs from 5.09% ± 1.02% to 17.35% ± 0.81%, reflecting antigen-driven maturation. IVNMs and Gel@IVNMs significantly reduced DC activation compared with Rapa, with the activated DC proportion decreasing to 7.99% ± 1.59% and 6.06% ± 0.63%, respectively, consistent with more effective modulation following enhanced uptake of IVNMs by DCs (Fig. 4E to G, Initial stimulation). Unlike the change in the tDC population, Rapa can directly inhibit inflammatory activation associated with DC maturation. This also appears to be the reason for the divergent changes in DCs and tDCs after initial stimulation. However, short-term inhibition of inflammation is not our goal.

We next examined the DC response to restimulation with CLAs, an important indicator of immune tolerance induction. After restimulation with CLAs, DCs exhibited a dramatically different response. The percentage of tDCs was significantly higher in the IVNM group than in the CLA group (*P* < 0.001) (Fig. 4E to G, Restimulation). The percentage of DCs with high PD-L1 expression increased from 12.06% ± 3.77% to 26.62% ± 2.08%; thus, the proportion of PD-L1^+^ cells nearly doubled, indicating that upon CLA restimulation, DCs rapidly up-regulated PD-L1 and acquired a tDC phenotype. The Gel@IVNMs group also differed significantly from the CLA group (*P* < 0.001), with the percentage of DCs with high PD-L1 expression increasing to 39.04% ± 1.05%, more than 3-fold the value in the CLA group and higher than that in the IVNM group. These results demonstrate that IVNMs, particularly when incorporated into Gel, strongly promote tolerogenic differentiation upon antigen reencounter. Furthermore, due to the incorporation of Gel, the tDC proportion was significantly higher in the Gel@IVNMs group than in the IVNM group (*P* < 0.01). This indicates that Gel enhances the efficacy of IVNMs in establishing immune tolerance (Fig. 4E to G, Restimulation). From the perspective of long-term effects, restimulation appeared to affect DC maturation similarly to the initial stimulation and had little additional effect on DCs that were already activated. The proportion of activated DCs did not significantly differ between first and second stimulation within each group, and the between-group trends remained unchanged from the first stimulation (Fig. 4F and G). These data suggest that IVNMs did not measurably alter the proportion of activated DCs under these conditions.

Mature DCs tend to secrete proinflammatory cytokines, whereas tDCs preferentially produce anti-inflammatory mediators. The expression levels of inflammatory factors examined by ELISA further confirmed the phenotypic results. The ELISA results showed that after restimulation, the levels of interferon-γ (IFN-γ) and IL-2 in the IVNMs and Gel@IVNMs groups decreased, while the expression levels of IL-10 and transforming growth factor-β (TGF-β) increased (Fig. 4H). The increased expression of IL-10 and TGF-β was a distinct characteristic of the tDC phenotype, which indirectly confirms the conclusion that tDC phenotype expression increases under restimulation. Different DC phenotypes also exhibit distinct cell sizes and morphologies, providing a complementary visual assessment. Thus, we evaluated the morphological changes of DCs during this process through cytoskeleton staining. After the initial stimulation and restimulation with CLAs, DCs displayed an irregular morphology with distinct dendritic features, characterized by numerous slender, branched dendritic protrusions extending from the cell surface, together with an increase in cell volume. In contrast, DCs in the restimulation groups of IVNMs and Gel@IVNMs were smaller and more nearly round or oval, with fewer and shorter dendritic protrusions, or even no obvious protrusions (Fig. S5). This is consistent with the above conclusions and allows for a more intuitive observation of DC changes.

### Gel@IVNMs suppresses T-cell activation, promotes CLAs-specific Treg responses, and limits inflammatory reactivation upon restimulation with CLAs

T cells directly activate inflammation and serve as the final endpoint during the establishment of immune tolerance. To monitor the impact of DC phenotypic changes on T cell response, DCs were cocultured with primary T cells and subjected to the same initial and restimulation procedures used for DCs (Fig. 5A). We analyzed the expression of CD127 in CD4^+^/CD25^+^ T cells using immunofluorescence and FC. CD127 expression serves as a key marker for distinguishing effector T cells from Tregs, as it is highly expressed in effector T cells but minimally or not expressed in Tregs [48,49].

**Fig. 5.**
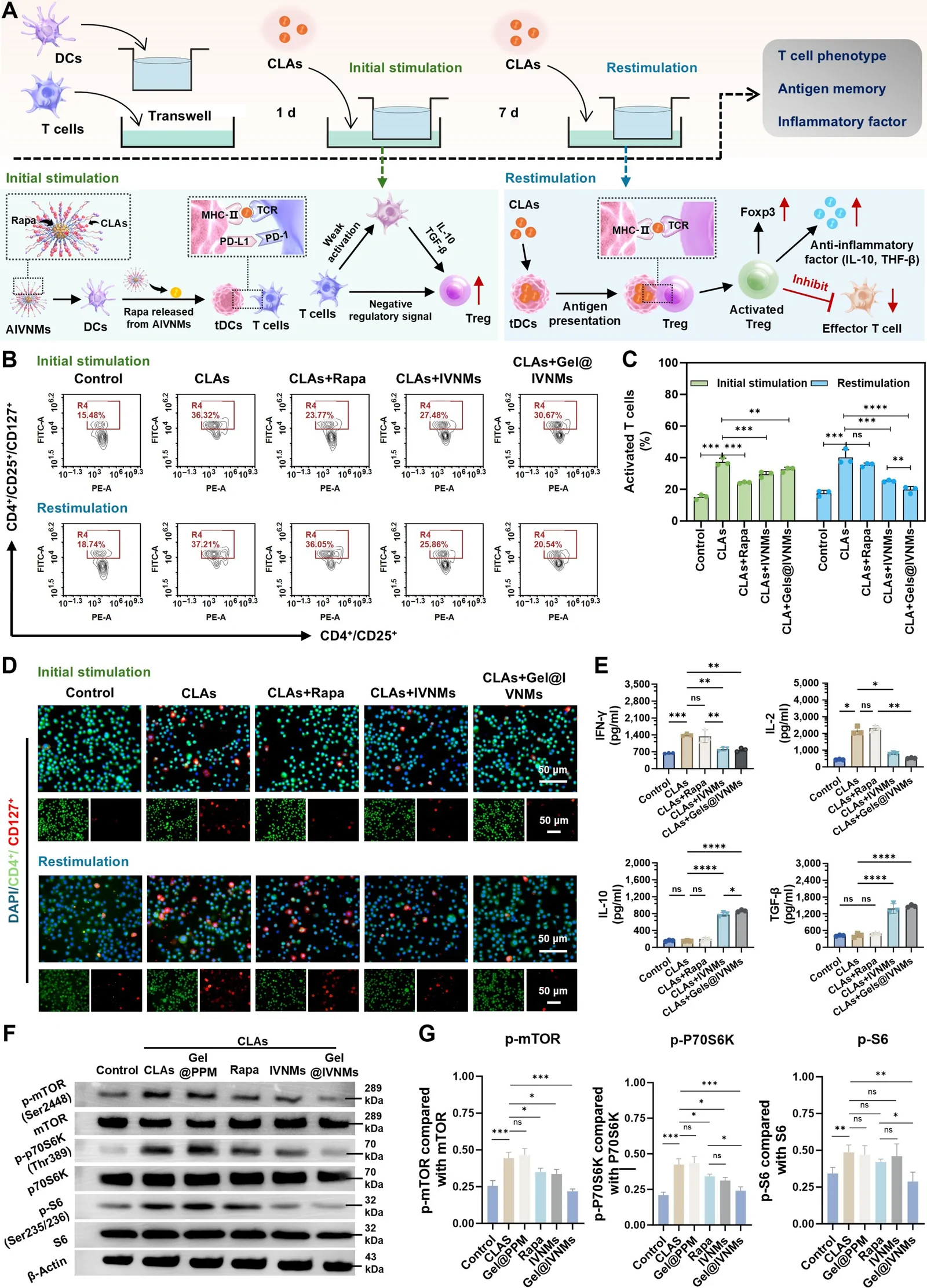
Phenotypic changes and inflammatory factor secretion of T cells stimulated by cardiac lysate antigens (CLAs). (A) Coculture protocol for dendritic cells (DCs) and T cells. The tolerogenic DCs (tDCs) present antigens to T cells; meanwhile, programmed death ligand 1 binds to programmed cell death protein 1 on T cells and inhibits T cell activation, promoting the phenotypic conversion of T cells to regulatory T cells (Tregs). Furthermore, due to the lack of activation signals such as CD80 and CD86, some T cells exhibit a weak activation state after receiving antigen presentation, and under the regulation of key factors such as interleukin-10 (IL-10) and transforming growth factor-β (TGF-β), they undergo phenotypic transition to Tregs. Under restimulation, tDCs phagocytose antigens and present them to Tregs. Tregs are rapidly activated, express high levels of Forkhead box P3 (FOXP3), and directly inhibit T cell activation, while secreting large amounts of anti-inflammatory factors. (B) Flow cytometry (FC) results for T cell phenotype. (C) Quantification of T cell phenotypes by FC. (D) T cell phenotype immunofluorescence staining results, where blue represents cell nuclei, red represents CD127, and green represents CD4. (E) Expression of interferon-γ (IFN-γ), IL-2, IL-10, and TGF-β after coculture of DCs and T cells. (F) Western blot analysis of the activation status of the mechanistic target of rapamycin (mTOR)-p70S6K-S6 pathway after treatment with Gel@PPM, Rapa, inverse vaccine nanomimics (IVNMs), and Gel@IVNMs under CLA stimulation. (G) The percentage of phosphorylation of mTOR, p70S6K, and S6 was statistically analyzed based on the gray values of the western blot results (compared to the unphosphorylated proteins). Data are presented as means ± SD, with individual points representing biological replicates. Statistical significance is indicated as ns, not significant; *P < 0.05; **P < 0.01; ***P < 0.001; ****P < 0.0001.

Similar to the changes in DCs, T cells are rapidly activated upon initial exposure to CLAs. After the initial stimulation, CLAs significantly increased CD127^+^ expression among CD4^+^/CD25^+^ T cells (37.31% ± 2.28%) compared to the control group (15.18% ± 1.51%), indicating robust effector T cell activation. Rapa exhibited a direct inhibitory effect on inflammatory activation to a certain extent [50]. Specifically, the Rapa group (24.29% ± 0.48%) and IVNM group (29.50% ± 1.81%) exhibited significantly reduced CD127 expression relative to CLAs, suggesting suppression of T cell activation. CD127 expression in the Gel@IVNMs group (32.45% ± 1.46%) was reduced compared with CLAs but to a lesser extent than Rapa (Fig. 5B and C). Immunofluorescence results were consistent with FC, showing intense CD127 signals in the CLA group and weaker signals in Rapa, IVNMs, and Gel@IVNMs groups (Fig. 5D). These differences were consistent with variation in Rapa exposure: Direct Rapa administration produced the strongest early inhibitory effect on T cells, whereas sustained-release Gel@IVNMs produced a weaker effect.

Upon restimulation, the T cell response reflects the state of immune tolerance and the potential for inflammatory reactivation. T cell responses differed markedly from those observed during the initial stimulation. The FC and immunofluorescence results showed that after restimulation, T cells exhibited robust activation and high CD127 expression, consistent with an effector T cell phenotype. Notably, under restimulation, CD127 expression showed no significant differences between the Rapa group and the CLA group, which contrasts with the clear differences observed during initial stimulation. These findings directly demonstrate that the inhibitory effect of Rapa on T cell activation, mediated through DC suppression, does not establish a memory immune response. Once Rapa was removed, its direct inhibitory influence was lost. When restimulated with CLAs, T cells in the Rapa group rapidly reactivated, with the proportion of CD127^+^ cells among CD4^+^/CD25^+^ populations accounting for 35.49% ± 4.10%, showing no significant difference from the CLA group (40.19% ± 4.57%). The IVNM and Gel@IVNMs groups exhibited a distinctly different response, showing a notable inhibitory state in response to CLA restimulation. In contrast, the proportion of CD4^+^/CD25^+^/CD127^+^ T cells in the IVNM group decreased significantly to 25.21% ± 0.57% and further to 19.69% ± 2.11% in the Gel@IVNMs group. Both proportions were significantly lower than that in the CLA group. Moreover, a significant difference was also observed between the IVNM and Gel@IVNMs groups, indicating that the incorporation of Gel enhances the tolerogenic efficacy of IVNMs, which is directly related to the improved drug release efficiency and sustained activity. Collectively, these results demonstrate that although Rapa directly suppresses T cell activation, it fails to induce immune tolerance memory. During the initial stimulation, IVNMs and Gel@IVNMs exhibited weaker T cell inhibitory activity compared to direct Rapa administration. However, in response to CLA restimulation, both the IVNM and Gel@IVNMs treatment groups showed a CLAs-responsive tolerogenic state consistent with immune memory and significantly suppressed T cell reactivation; incorporation into Gel further enhanced this effect (Fig. 5B to D).

Changes in cytokine secretion provide an additional indicator of T cell phenotype. Based on the results of T cell phenotype analysis, we performed quantitative analysis of cytokines secreted by T cells. The results were consistent with the changes in T cell phenotype. After restimulation, the levels of proinflammatory cytokines IFN-γ and IL-2 increased substantially in the CLA groups, whereas both the IVNM and Gel@IVNMs groups showed significant reductions. In contrast, IL-10 and TGF-β showed the opposite trend: The CLAs and Rapa groups exhibited similar expression levels comparable to the Control group, with no significant statistical differences, while both the IVNM and Gel@IVNMs groups showed a significant increase. Like the cytokine trends in DCs, these results cannot distinguish whether the changes are due to altered cytokine expression in T cells or DCs (Fig. 5E). Taken together, in our in vitro studies, we verified the antigen-capture capacity of IVNMs and their enhanced uptake by DCs in vitro. Under CLA stimulation, we achieved controllable phenotypic modulation of both DCs and T cells. Furthermore, after long-term immune tolerance training mediated by the sustained release characteristics of Gel, restimulation with CLAs led to a pronounced phenotypic shift in DCs and T cells, including the emergence of distinct tDCs. This outcome aligns with our therapeutic strategy: The induced tDCs and T cells exhibit a memory effect that enables specific immune tolerance to CLAs, leading to reduced T cell activation and decreased proinflammatory cytokine expression.

To further investigate how IVNMs affect T cell signaling, we examined the protein expression levels of phosphorylated mechanistic target of rapamycin (p-mTOR) (Ser2448), mTOR, p-p70S6K (Thr389), p70S6K, p-S6 (Ser235/236), and S6 in each group. The ratios of phosphorylated protein to corresponding total protein (p-mTOR/mTOR, p-p70S6K/p70S6K, and p-S6/S6) were calculated to assess the activation status of the mTORC1 signaling pathway.

Compared with the Control group, the CLA group showed a marked increase in p-mTOR levels and the p-mTOR/mTOR ratio, with mTOR phosphorylation rising to 45.28% ± 3.27%. In contrast, the Rapa group significantly inhibited mTOR phosphorylation, reducing the p-mTOR/mTOR ratio to 34.63 ± 2.17%. The Gel@IVNMs group also exhibited a notable decrease in mTOR phosphorylation, with the p-mTOR/mTOR ratio declining from 26.16% ± 3.77% in the control group to 21.94% ± 1.25%. Notably, no significant differences in total mTOR protein expression were observed among the groups (Fig. 5F and G). p70S6K is a direct downstream substrate of mTORC1, and its phosphorylation at Thr389 directly reflects mTORC1 kinase activity [6,46]. Consistent with the changes in mTOR, CLA stimulation significantly increased p-p70S6K levels, whereas Rapa treatment partially reduced p70S6K phosphorylation. Both IVNMs and Rapa exhibited a limited inhibitory effect on p70S6K. Notably, Gel@IVNMs significantly decreased p70S6K phosphorylation (Fig. 5F and G). As a downstream effector of p70S6K, S6 phosphorylation further corroborates the activation status of the pathway [47,48]. Compared with the Control group, the CLA group displayed a significantly increased p-S6/S6 ratio. The Rapa group profoundly suppressed S6 phosphorylation, resulting in a markedly reduced ratio. Similarly, the Gel@IVNMs group exhibited a significantly decreased ratio. Total S6 protein levels remained unchanged across all groups (Fig. 5F and G). Collectively, compared with the Control group, Rapa, IVNMs, and Gel@IVNMs significantly reduced the phosphorylation levels of p-mTOR (Ser2448), p-p70S6K (Thr389), and p-S6 (Ser235/236). Among these treatments, Gel@IVNMs exerted the most prominent inhibitory effect on the mTOR pathway. Moreover, total protein expression levels were not substantially affected, suggesting that treatment with IVNMs may contribute to immune tolerance induction by suppressing the activity of the mTORC1 signaling pathway.

### Therapeutic effects of Gel@IVNMs after intramyocardial injection in a rat MI model

To evaluate the efficacy of Gel@IVNMs in inducing immune tolerance in vivo, its regulatory effect on inflammation, and its impact on cardiac function after MI, we conducted in vivo experiments in rats. First, we confirmed the biosafety of the nanocomposite Gel via blood biochemical indicators and hematoxylin and eosin (H&E) staining of major organs (Fig. S6 and Table S2). The overall timeline of the animal experiment is illustrated in Fig. 6A. MI was induced in rats via ligation of the left anterior descending (LAD) coronary artery, followed by intramyocardial injection of Gel@IVNMs for treatment. Organ imaging revealed that Gel markedly prolonged the retention time of IVNMs in the heart, thereby supporting sustained local immunomodulation. At 14 d postinjection, the Gel@IVNMs group still retained 50.33% ± 1.95% signal intensity in the heart, significantly higher than the IVNM group (9.63±3.23%, Fig. 6B and C).

**Fig. 6.**
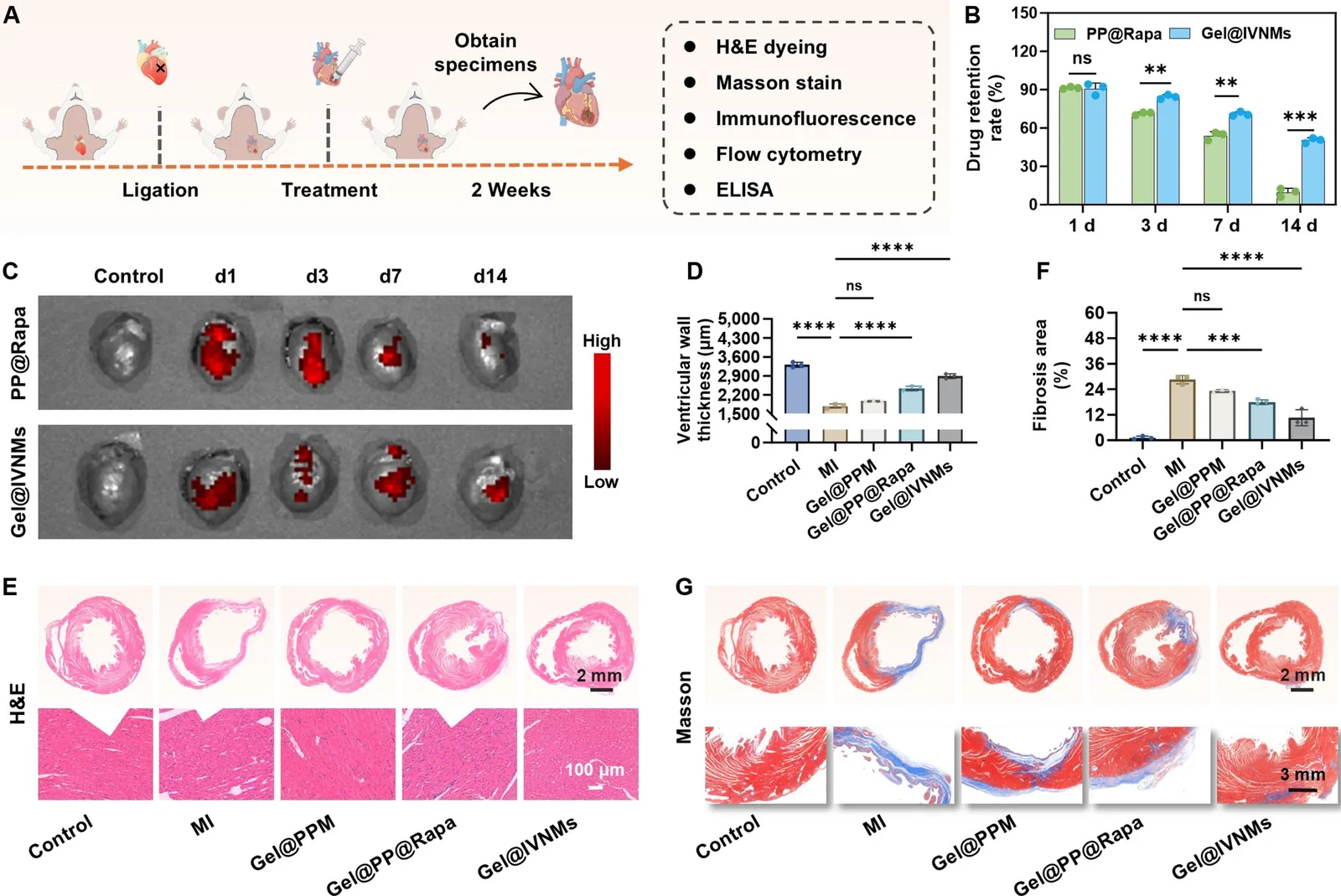
Animal experiment procedures, heart preservation time, and changes in heart structure. (A) Animal experiment protocol and testing items. (B and C) Retention time and statistical results of inverse vaccine nanomimics (IVNMs) and Gel@IVNMs after intramyocardial injection. (D and E) Hematoxylin and eosin (H&E) staining results of heart sections and statistical results of ventricular wall thickness. (F and G) Masson staining of myocardial fibrosis levels and statistical results of fibrosis area percentage. Data are presented as means ± SD, with individual points representing biological replicates. Statistical significance is indicated as ns, not significant; **P < 0.01; ***P < 0.001; ****P < 0.0001.

We comprehensively evaluated changes in cardiac structure. Overall, the therapeutic strategy of Gel@IVNMs attenuated the deterioration of cardiac structure after MI, including preservation of ventricular wall thickness and a significant reduction in myocardial fibrosis levels. These beneficial structural changes were conducive to cardiac recovery and preservation of cardiac function following MI. Specifically, H&E and Masson staining were performed. In the MI group, CMs in the infarcted region displayed extensive necrosis, edema, and cytolysis, with disruption and loss of myocardial architecture. Compared with the control group (3,307.00 ± 93.47 μm), ventricular wall thickness in the MI group was significantly reduced (1,786.33 ±77.68 μm), accompanied by expanded interstitial spaces and massive inflammatory infiltration. In the Gel@PPM group, partial restoration of the ventricular wall was observed. This may be attributed to the mechanical support provided by Gel to the heart, thereby improving myocardial structure. In contrast, the Gel@IVNMs group exhibited a markedly improved myocardial structure, showing compact tissue organization resembling that of the control group, attributed to the immunoregulatory action of Rapa (Fig. 6D and E).

The deposition of collagen fibers in the MI area was observed by Masson staining to evaluate the degree and scope of fibrosis. In the MI group, the central infarcted area showed dense, blue-stained fibrotic connective tissue with sparse, red-stained myocardium. Around the infarcted area, connective tissue and myocardial fibers were alternately distributed, and the ventricular wall was markedly thinned. In contrast, the degree of collagen deposition in all treatment groups was reduced compared with the MI group. Notably, the Gel@IVNMs group showed dense cellular organization with minimal visible collagen deposition, indicating that Gel@IVNMs effectively attenuated post-MI myocardial fibrosis (Fig. 6F and G).

To evaluate apoptosis in cardiac tissue, we performed terminal deoxynucleotidyl transferase deoxyuridine triphosphate nick-end labeling staining (green) as shown in Fig. S6. Given that excessive CM apoptosis is a key pathological feature of MI-induced myocardial damage, quantifying apoptotic cells provides direct insight into the protective efficacy of each formulation against ischemic injury. Relative to the control group (3.59% ± 0.33%), the MI group showed a marked increase in apoptotic cells to 12.54% ± 0.83%, a nearly 3.5-fold increase relative to the control reflecting the severe myocardial injury caused by ischemia and subsequent inflammation. The Gel@PPM group slightly reduced the proportion of apoptotic cells, which may reflect the stabilizing effect of Gel on the local microenvironment. In contrast, the Gel@PP@Rapa and Gel@IVNMs groups, both incorporating Rapa, markedly alleviated the inflammatory microenvironment within the infarct region and conferred substantial myocardial protection, reducing apoptosis to 5.58% ± 0.16% and 4.57% ± 0.17%, respectively.

Post-MI, α-smooth muscle actin (α-SMA) and platelet endothelial cell adhesion molecule-1 (CD31) serve as key markers for assessing cellular responses and angiogenesis in the damaged myocardium [51,52]. Specifically, α-SMA is a hallmark of vascular smooth muscle cells involved in vessel maturation, while CD31 is a classic endothelial cell marker that reflects neovascularization density making their combined detection ideal for evaluating myocardial repair and neovascularization efficacy. Immunofluorescence staining of α-SMA and CD31 in the infarct area was used to evaluate myocardial repair and neovascularization. Compared with the MI group, all treatment groups demonstrated a significant increase in neovascularization, attributable to the 3-dimensional scaffold support provided by Gel, which fosters vascular formation and creates a favorable local microenvironment. Among all groups, the Gel@IVNMs treatment group exhibited the strongest positive signals, indicating the highest density of newly formed blood vessels and a pronounced proangiogenic effect in myocardial tissue. Collectively, these results support a cardioprotective effect of Gel@IVNMs in vivo (Fig. 7A and B).

**Fig. 7.**
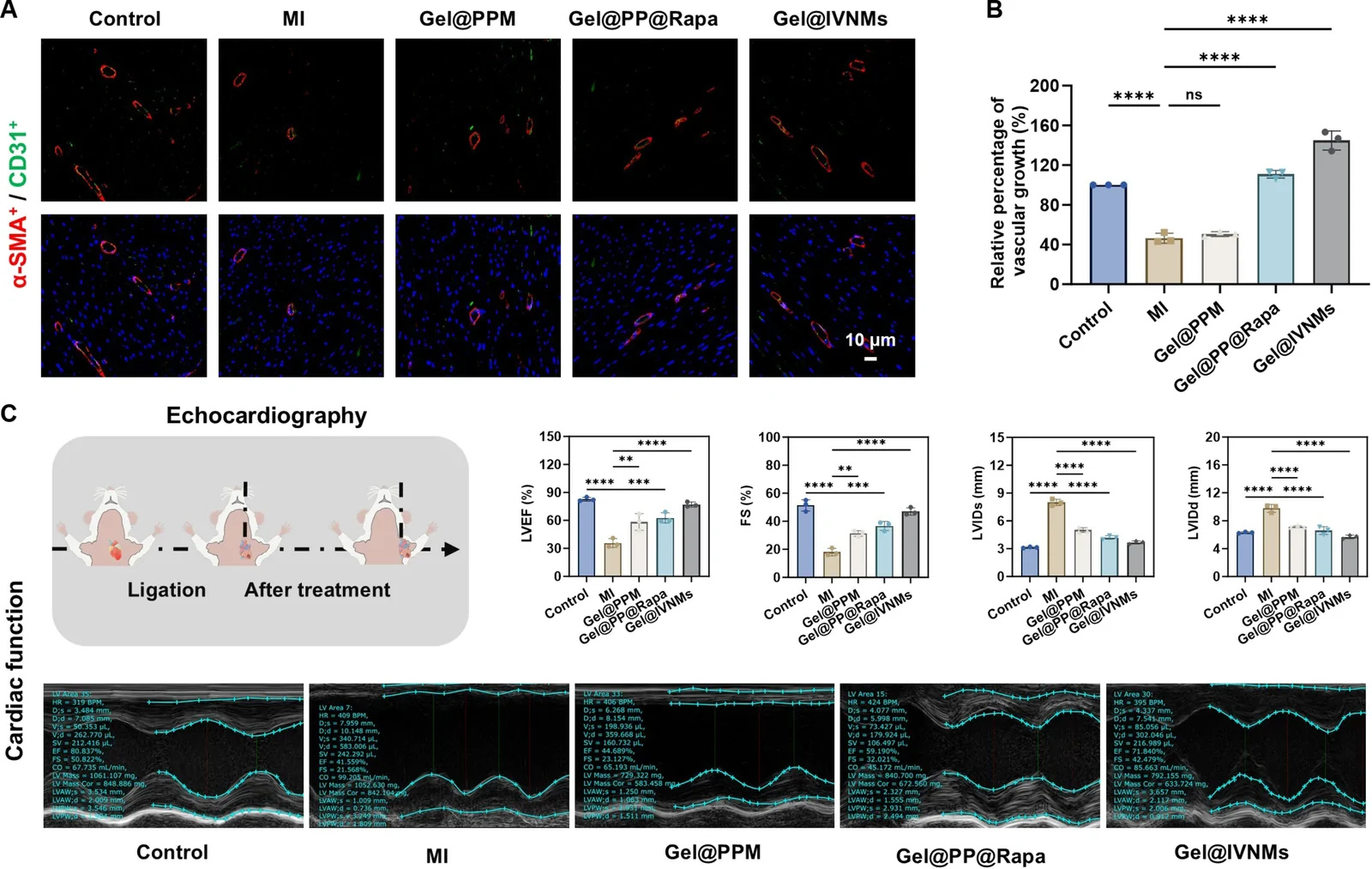
Changes in vascular growth and cardiac pumping function. (A and B) Immunofluorescence staining and statistical results of vascular growth in heart sections. (C) Echocardiography detection results and statistical results of LVEF, fractional shortening (FS), left ventricular internal diameter at end-systole (LVIDs), and left ventricular internal diameter at end-diastole (LVIDd). Data are presented as means ± SD, with individual points representing biological replicates. Statistical significance is indicated as ns, not significant; **P < 0.01; ***P < 0.001; ****P < 0.0001.

To further evaluate therapeutic outcomes, rats were anesthetized for echocardiographic examination, and key indices of cardiac function were statistically analyzed. The left ventricular ejection fraction (LVEF) and fractional shortening (FS) assess cardiac contractility by measuring the proportion of blood ejected per cardiac cycle and the degree of left ventricular diameter reduction during systole, respectively. Additionally, the left ventricular internal diameter at end-diastole and end-systole can be used to indicate impaired systolic performance. As expected, the LVEF and FS of the MI group were lower than those of the control group, indicating the successful establishment of the MI model. Compared with the MI group, the rats in the Gel@IVNMs group showed significantly increased LVEF and FS and significantly decreased left ventricular internal diameter at end-diastole and left ventricular internal diameter at end-systole, demonstrating effective restoration of cardiac contractile function, mitigation of myocardial damage, and promotion of functional recovery (Fig. 8C and Fig. S8).

**Fig. 8.**
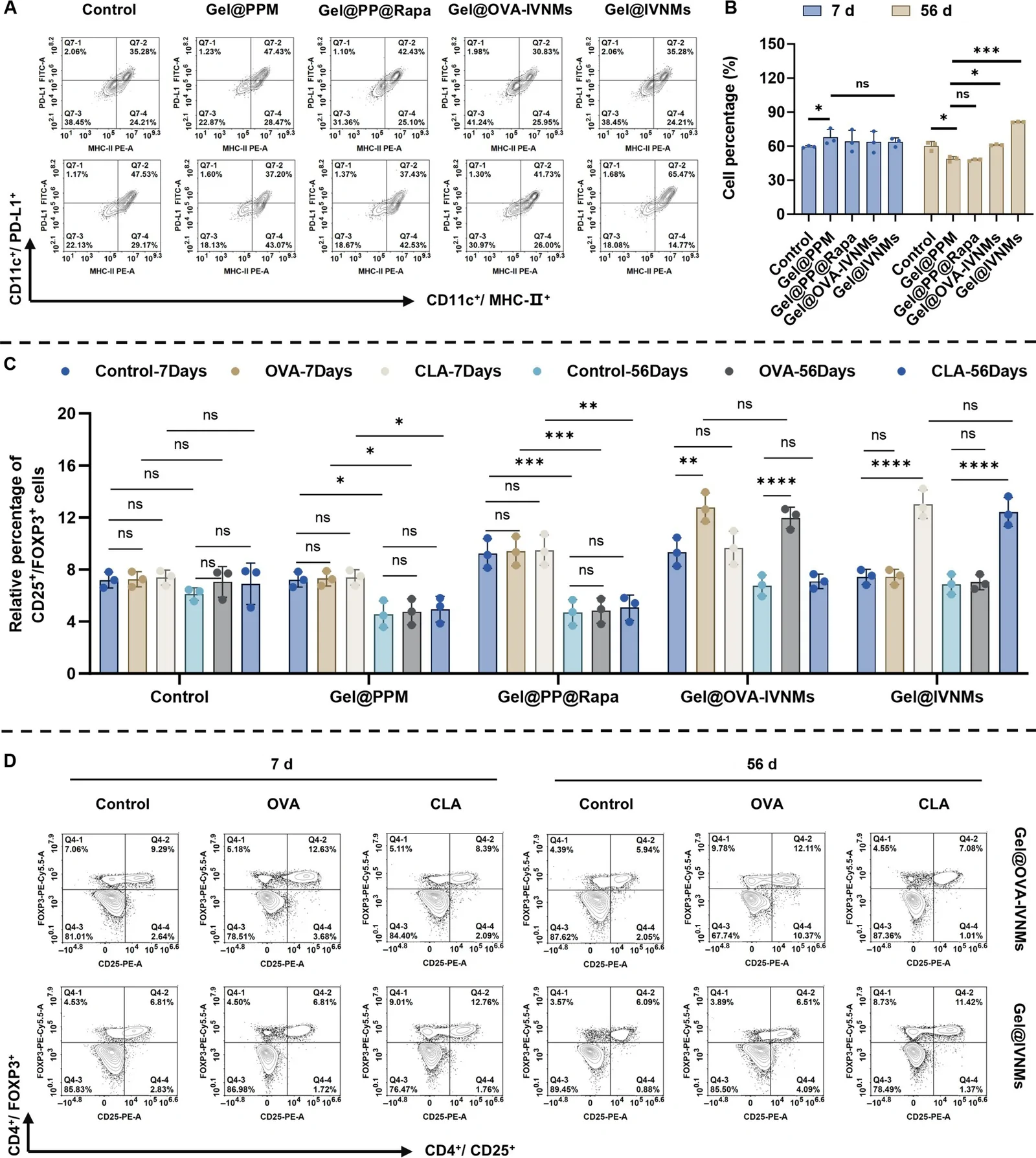
Changes in dendritic cell phenotype and regulatory T cell responses in cardiac-draining lymph nodes after myocardial infarction (MI). (A) Representative flow cytometry plots showing CD11c^+^MHC-II^+^ dendritic cells and programmed death ligand 1 (PD-L1) expression in the Control, Gel@PPM, Gel@PP@Rapa, Gel@OVA-IVNMs, and Gel@IVNMs groups at 7 and 56 d after MI. (B) Quantification of the proportion of PD-L1^+^ cells within the CD11c^+^MHC-II^+^ dendritic cell population at 7 and 56 d. (C) Quantification of CD4^+^CD25^+^FOXP3^+^ regulatory T cells (Tregs) in lymph node-derived cells from the 5 treatment groups after 48 h of culture under unstimulated Control, OVA, or cardiac lysate antigen (CLA) stimulation at 7 and 56 d. (D) Representative flow cytometry plots of CD25 and Forkhead box P3 (FOXP3) expression within CD4^+^ T cells in the Gel@OVA-IVNMs and Gel@IVNMs groups following Control, OVA, or CLA stimulation at 7 and 56 d. Data are presented as means ± SD, with individual points representing biological replicates. Statistical significance is indicated as ns, not significant; *P < 0.05; **P < 0.01; ***P < 0.001; ****P < 0.0001.

Evaluation of cardiac structure and function over 2 weeks showed that Gel@IVNMs protected the heart against post-MI injury during the initial observation period. To determine whether this benefit was accompanied by durable immune regulation, we revised the grouping scheme, extended the in vivo follow-up to 56 d, and assessed DC phenotypes in the anterior mediastinal lymph nodes. CD11b/c and major histocompatibility complex-II (MHC-II) were used to identify the principal DC population, and the proportion of PD-L1^+^ cells within the CD11b/c^+^MHC-II^+^ population was quantified, this subset was operationally classified as tDCs. At the early post-MI time point (day 7), the Gel@PPM group and all Rapa-containing groups (Gel@PP@Rapa, Gel@OVA-IVNMs, and Gel@IVNMs) showed higher tDC proportions than the Control group. This early increase was consistent with activation of repair-associated immune responses after tissue injury. The tDC proportions were 67.70% ± 7.14% in the Gel@PPM group and 64.23% ± 9.77% in the Gel@PP@Rapa group, indicating that the addition of Rapa did not significantly alter the tDC proportion at this time point. Similarly, the values for the Gel@OVA-IVNMs group (63.53% ± 9.49%) and Gel@IVNMs group (63.52% ± 3.76%) did not differ significantly from that for the Gel@PPM group, although their mean values were slightly lower. Thus, any early effect of Rapa on the DC phenotype was insufficient to reach statistical significance under the tested conditions (Fig. 8A and B). By day 56, however, the flow-cytometric profile of DC phenotypes differed from that observed on day 7. The Control group showed no clear time-dependent change in tDC proportion, arguing against a major contribution from the sham procedure or external variation. In the Gel@PPM group, the tDC proportion decreased to 48.70% ± 2.26%, which was significantly lower than that in the day-56 Control group (60.24% ± 3.77%). After the early repair phase, the decline in this tolerogenic phenotype, even below the Control level, was associated with persistent inflammatory activation [53]. The absence of a significant change in the proportion of MHC-II^+^ cells among CD11b/c^+^ cells further suggested that the difference reflected phenotype rather than a broad change in this cell population. The Gel@PP@Rapa group showed a similar pattern, with the tDC proportion decreasing to 47.76% ± 0.81%. Once the initial Rapa exposure had subsided, its long-term effect on the DC phenotype did not differ significantly from that of the drug-free Gel@PPM control. In contrast, Gel@IVNMs increased the tDC proportion to 81.27% ± 0.35%, a highly significant difference from the Control group. Together with the structural and functional improvements observed at day 14, these findings indicate that the beneficial effects of Gel@IVNMs emerged during the early post-MI period and were accompanied by sustained immunoregulatory changes at day 56.

The change in DC phenotype supports an immunomodulatory effect of Gel@IVNMs after MI but does not by itself establish that the therapeutic benefit results from immune tolerance. We therefore evaluated T cell responses to antigen restimulation early point (day 7) and a late time point (day 56) and assessed Treg proportions under different antigen conditions. Single-cell suspensions from the anterior mediastinal lymph nodes were cultured without antigen (Control-7Days and Control-56Days conditions) or restimulated with the irrelevant antigen ovalbumin (OVA) or autologous CLAs prepared from the same rat. The complete flow cytometry data are shown in Fig. S9. T cell phenotypes were evaluated by CD4, CD25, and Forkhead box P3 (FOXP3) expression, and CD4^+^CD25^+^FOXP3^+^ cells were defined as Tregs. We focused on comparing the Gel@OVA-IVNMs and Gel@IVNMs groups under the different ex vivo antigen-restimulation conditions. In the Control group, Treg proportions did not differ significantly among the unstimulated, OVA-stimulated, and CLAs-stimulated conditions at either day 7 or day 56, indicating minimal antigen-dependent variation in untreated animals. Within the Gel@PPM group, neither OVA nor CLA restimulation caused a clear change in Treg proportion at either time point; however, the overall Treg proportion was lower at day 56 than at day 7. This pattern is consistent with resolution of the early post-MI repair response and paralleled the change in DC phenotype, with Treg proportions returning toward the basal level by day 56. The Gel@PP@Rapa group showed a similar temporal pattern, with generally higher Treg proportions early and lower proportions at day 56. Rapa treatment was associated with a higher early Treg proportion, suggesting that, although Rapa did not significantly alter the early DC phenotype, it affected the T cell response, consistent with its anti-inflammatory activity.

Notably, comparison of the Gel@OVA-IVNMs and Gel@IVNMs groups demonstrated a formulation-matched Treg response that depended on the antigen loaded onto the IVNMs. In the Gel@OVA-IVNMs group, OVA restimulation produced a highly significant increase in the Treg proportion relative to the unstimulated condition at both time points. OVA restimulation increased the Treg proportion to 12.77% ± 1.11% on day 7 and 11.97% ± 0.82% on day 56. Although Treg proportions after OVA restimulation did not differ significantly between days 7 and 56, the proportion under unstimulated conditions was significantly lower on day 56 than on day 7. The persistence of the OVA-associated increase therefore indicates retention of an antigen-responsive Treg response over the long term. In the Gel@IVNMs group, the Treg response was more pronounced following CLA restimulation, which increased the Treg proportion to 13.02% ± 1.12% at the early time point and 12.42% ± 1.11% at the late time point. These findings support an antigen-associated, formulation-matched Treg recall response consistent with the development of immune tolerance to the captured antigen; however, they do not by themselves exclude contributions from broader immunomodulation (Fig. 8C and D).

To assess changes in inflammatory cell infiltration during the early and late phases of treatment, rat cardiac sections were immunostained for macrophage markers (CD68, green; inducible nitric oxide synthase [iNOS], red; and CD206, purple). Within the CD68^+^ population, macrophages with high iNOS or CD206 expression were operationally classified as M1-like proinflammatory or M2-like reparative/anti-inflammatory phenotypes, respectively [54]. At the early post-MI time point, all drug-containing treatment groups showed a lower proportion of iNOS-high macrophages and a higher proportion of CD206-high macrophages than the drug-free Gel@PPM group, with statistically significant differences. This pattern was consistent with early suppression of inflammation in the drug-containing groups. In view of the similar responses across the Rapa-containing groups, the early phenotype was consistent with a Rapa-associated effect. However, no significant differences in either the iNOS-high or CD206-high macrophage proportion were detected among the Gel@PP@Rapa, Gel@OVA-IVNMs, and Gel@IVNMs groups at this time point (Fig. 9A and B). Early ELISA measurements supported the same interpretation: IL-2, IFN-γ, TGF-β, and IL-10 levels were comparable among these groups and followed similar directional trends (Fig. 9C).

**Fig. 9.**
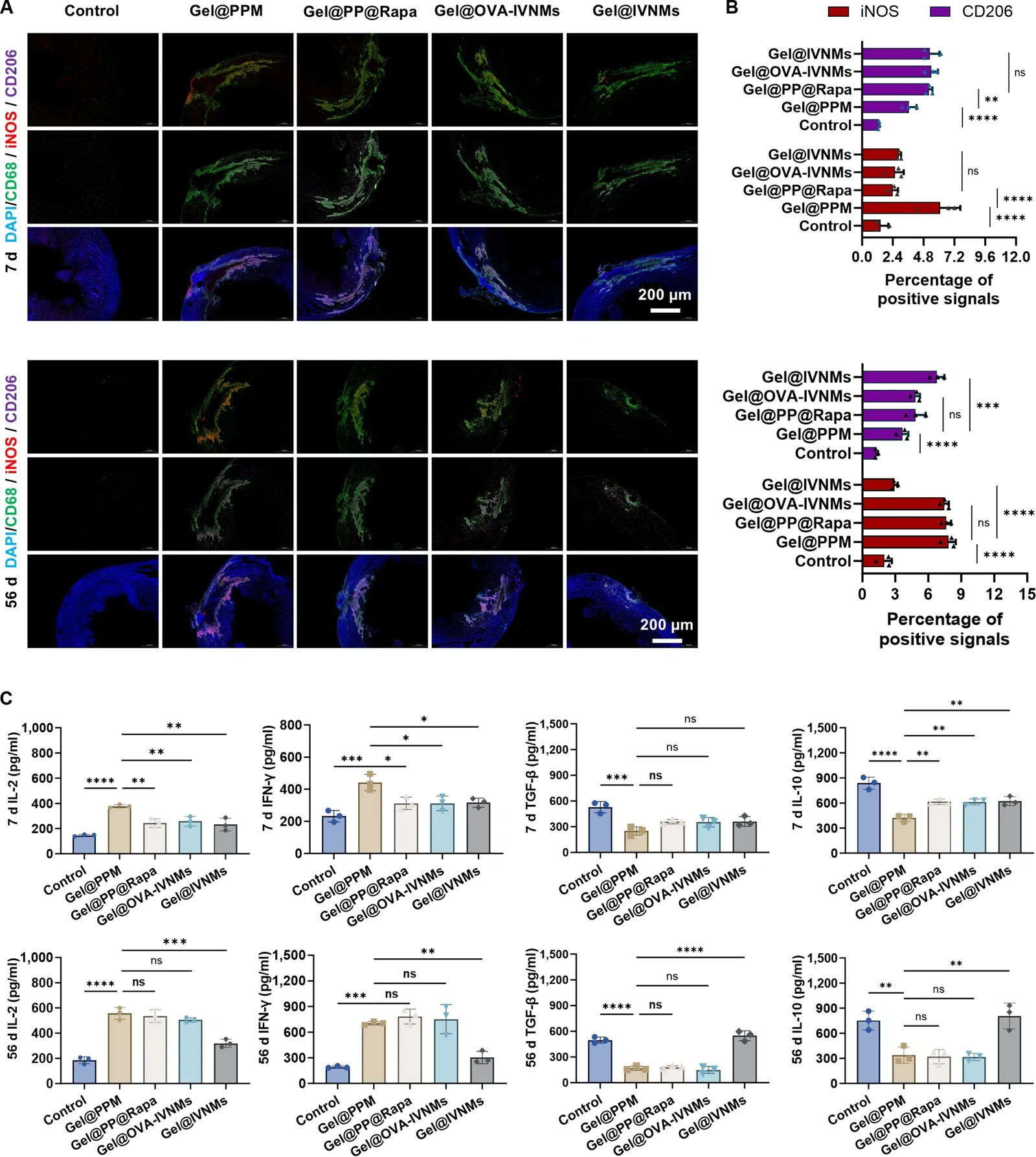
Changes in myocardial macrophage phenotypes and inflammatory cytokine profiles after myocardial infarction (MI). (A) Representative immunofluorescence images of heart sections from the Control, Gel@PPM, Gel@PP@Rapa, Gel@OVA-IVNMs, and Gel@IVNMs groups at 7 and 56 d after MI. Nuclei were stained with 4′,6-diamidino-2-phenylindole (DAPI; blue), macrophages with CD68 (green), proinflammatory macrophage-associated inducible nitric oxide synthase (iNOS) with red fluorescence, and reparative/immunomodulatory macrophage-associated CD206 with purple fluorescence. Scale bars, 200 μm. (B) Quantification of iNOS- and CD206-positive signals in myocardial sections at 7 and 56 d after MI. (C) Enzyme-linked immunosorbent assay quantification of interleukin-2 (IL-2), interferon-γ (IFN-γ), transforming growth factor-β (TGF-β), and IL-10 levels in myocardial tissue at 7 and 56 d after MI. Data are presented as means ± SD, with individual points representing biological replicates. Statistical significance is indicated as ns, not significant; *P < 0.05; **P < 0.01; ***P < 0.001; ****P < 0.0001.

At the late treatment time point, after the initial effect of Rapa had subsided, the Gel@PP@Rapa and Gel@OVA-IVNMs groups showed inflammatory profiles similar to that of the drug-free Gel@PPM group. This pattern suggests that Rapa alone provided transient rather than sustained inflammatory control after the initial exposure had ended. In contrast, the Gel@IVNMs group retained a marked anti-inflammatory profile. The proportion of iNOS-high macrophages decreased from 7.84% ± 0.64% in the Gel@PPM group to 2.92% ± 0.29% in the Gel@IVNMs group. Although this value remained above the Control level, it was highly significantly lower than that in the Gel@PPM group. Meanwhile, the proportion of CD206-high macrophages increased from 3.67% ± 0.52% in the Gel@PPM group to 6.80% ± 0.66% in the Gel@IVNMs group. These findings indicate a durable shift toward a reparative macrophage phenotype after Gel@IVNMs treatment (Fig. 9A and B). The day-56 ELISA results were consistent with the immunofluorescence findings: IL-2 and IFN-γ levels were lower, whereas TGF-β and IL-10 levels were higher, in the Gel@IVNMs group. The other treatment groups did not differ significantly from the Gel@PPM group. Together, these findings are consistent with sustained inflammatory control in the Gel@IVNMs group (Fig. 9C).

## Conclusion

In this study, we designed IVNMs with in situ antigen-capture capacity and mannose-facilitated uptake by CD206-expressing immune cells, which, when combined with a pH-responsive, degradable Gel, enable long-term immune tolerance induction for post-MI recovery therapy. These IVNMs can effectively capture antigens released by necrotic CMs and regulate the phenotypic transition of DCs to tDCs, thereby increasing the proportion of CLAs-responsive Tregs. Consequently, this treatment reduces CM apoptosis, alleviates myocardial fibrosis, promotes angiogenesis, and improves cardiac function.

We synthesized polymers with both hydrophilic and hydrophobic ends and customized the polymer length and the terminal groups of the hydrophilic ends to provide antigen-capture capacity and promote mannose-facilitated cellular uptake. Antigen adsorption experiments showed that polymers with maleimide-terminated chains could efficiently adsorb free protein antigens in the environment. This is because maleimide forms covalent bonds with thiol groups in proteins via Michael addition [55]. Protein quantification showed that the antigen protein in the environment after adsorption by IVNMs was significantly reduced (Fig. 2F to H). For the Schiff-base-cross-linked Gel, swelling and degradation differed significantly between pH 7.4 and 6.5. The slightly acidic environment was conducive to the gradual degradation of Gel and the release of IVNMs (Fig. 3H to J).

In vitro experiments characterized changes in DCs and T cell phenotypes after stimulation with CLAs and treatment with IVNMs, which constitutes the core mechanism of this study. Figure 4B to D shows that mannose modification at the chain ends of IVNMs enhanced their uptake by DCs under the tested in vitro conditions. Under the primary and secondary stimulation of CLAs, DCs were activated; however, treatment with IVNMs down-regulated the expression of CD80 and CD86 on the surface of DCs, inhibited DC activation, and promoted a tDC phenotype (Fig. 4E to G). tDCs present antigens to T cells; meanwhile, PD-L1 expressed at high levels on tDCs binds to the programmed cell death protein 1 receptor on T cells and inhibits T cell activation. This effect is manifested by the low expression of CD4 and CD127 (Fig. 6B to D) and the high secretion of anti-inflammatory factors such as IL-10 and TGF-β (Fig. 6E). Upon restimulation with CLAs, tDCs respond rapidly (with PD-L1 expression increasing from 12.06% ± 3.77% to 26.62% ± 2.08%) (Fig. 4E to G) and present antigens to Tregs, thereby inhibiting the sustained activation of T cells. This is reflected by a rapid increase in the proportion of Treg cells (Fig. 6B to D) and the rapid and massive secretion of anti-inflammatory factors (Fig. 6E).

In studies related to MI, researchers have established immune tolerance toward allogeneic antigens by injecting nanoparticles containing allogeneic antigens into the infarct site. However, the immune tolerance established using allogeneic antigens and a single injection lacks specificity and long-term efficacy [36]. In the present study, we injected hydrogels containing IVNMs into the infarct site. After 2 weeks of treatment, we observed that the levels of tDCs (Fig. 7A and B) and Treg cells (Fig. 7C to D) in the spleen and heart were increased, which in turn regulated the phenotype of macrophages and induced their transition to the anti-inflammatory M2 phenotype (Fig. 7E and F). This was accompanied by a decrease in the expression of proinflammatory factors and an increase in the expression of anti-inflammatory factors (Fig. 7G).

On this basis, we observed a marked improvement in cardiac structure in the rat MI model, along with a reduction in the level of CM apoptosis (Fig. S8A and B), an alleviation of myocardial fibrosis (Fig. 6G), the occurrence of marked angiogenesis at the infarct site (Fig. 8A and B), and an improvement in cardiac contractile function (Fig. 8C to G).

Immune tolerance induction has emerged as a powerful strategy to control pathological inflammation [29]. Current clinical evidence suggests that the early phase following an acute cardiovascular event may represent a therapeutic window for immune intervention; in patients with acute MI or acute heart failure, early in-hospital influenza vaccination is associated with improved clinical outcomes, whereas simply increasing the vaccine antigen dosage yields no additional benefit. These findings indicate that the benefits observed after acute myocardial injury may not stem from nonspecific immune stimulation, but rather from the timing of the intervention and the remodeling of immune response patterns. However, its application in MI has been constrained by limited antigen specificity, transient efficacy, and the requirement for predefined antigens. Previous inverse vaccine approaches relied on exogenously supplied or allogeneic antigens, resulting in incomplete tolerance induction and poor durability [56,57]. In contrast, the platform described here establishes immune tolerance directly within the infarct microenvironment by capturing endogenous antigens released from necrotic CMs, thereby ensuring antigen relevance, safety, and specificity.

A key feature of this system is its ability to induce immune tolerance memory rather than transient immunosuppression. Through sustained local presentation of autologous antigens and modulation of DC phenotype following cellular uptake, the IVNMs program a durable regulatory immune state that actively suppresses inflammatory reactivation upon antigen reencounter. Accordingly, the long-term tolerance findings should not be interpreted as evidence of exclusive particle uptake by DCs, and contributions from other CD206-expressing immune cells cannot be excluded. This distinguishes the present strategy from conventional anti-inflammatory or immunosuppressive therapies, which lack memory and require continuous administration [58,59].

Beyond MI, this in situ tolerance-induction paradigm is broadly applicable to diseases driven by excessive or recurrent immune activation following tissue injury, including ischemic, autoimmune, and inflammatory disorders [32,60]. By coupling endogenous antigen capture with spatiotemporally controlled immunomodulation, this work provides a generalizable framework for engineering immune tolerance without prior antigen knowledge, advancing the translational potential of inverse vaccines for precision immunotherapy.

Although some success has been achieved in treating small animals, the specific protein components within CLAs that drive immune tolerance induction remain unclear, and variations in antigen composition among individuals or disease states may influence treatment efficacy. Future studies should identify the key tolerogenic antigens and determine whether their abundance or immunogenicity correlates with therapeutic responses. From a clinical perspective, major translational barriers include standardizing the physicochemical properties and antigen-capture capacity of Gel@IVNMs, establishing reproducible large-scale manufacturing and quality-control procedures, optimizing the administration strategy, and comprehensively evaluating local and systemic immune safety. These issues must be addressed before the clinical applicability of this platform can be fully established.

## Materials and Methods

### Materials

The sources of reagents, consumables, and equipment used in this study are listed in Tables S3 and S4 in the Supplementary Materials. Throughout Materials and Methods, reagents and equipment are reported in the format (catalog number or model, brand, country); when no catalog number is applicable, the model or supplier identification is provided. The synthesis scheme for micellar polymers is presented in the Supplementary Materials.

### Synthesis of functionalized PEG-PCL derivatives and fabrication and characterization of IVNMs

To synthesize PCL, redistilled ε-caprolactone (10 g, 87.72 mmol), benzyl alcohol (278 mg, 2.57 mmol), and stannous chloride (300 mg, 3% of the total mass) were added into a 100-ml flask equipped with a vacuum stopcock. The mixture was dried under vacuum at 35 °C for 5 h and then heated to 140 °C for polymerization for 6 h. After cooling to room temperature (RT), 6 ml of dichloromethane (DCM) was added to dissolve the product overnight. The resulting solution was added dropwise to excess ice-cold ethanol with stirring to precipitate the product. The precipitate was filtered and vacuum-dried to obtain white powdered PCL.

For the synthesis of COOH-PEG-COOH, PEG (10 g, 5 mmol, *Mw* = 2,000, *Mw* = 4,000), succinic anhydride (1.5 g, 15 mmol), and sodium acetate (2.46 g, 30 mmol) were dissolved in 50 ml of dry DCM and reacted at RT until PEG was completely consumed, as monitored by thin-layer chromatography. The mixture was filtered, and 5 ml of 0.1 mol/l hydrochloric acid solution was added for extraction. The organic phase was then washed with saturated sodium chloride solution, separated, and dried overnight with anhydrous sodium sulfate (Na_2_SO_4_). After filtration to remove Na_2_SO_4_, the solvent was concentrated under reduced pressure to 10 ml and then added dropwise to stirred ice-cold ether to precipitate the product. The precipitate was filtered and vacuum-dried to obtain white powdered COOH-PEG-COOH.

To synthesize PP2000-COOH, COOH-PEG-COOH (4.00 g, 1.81 mmol) was dissolved in 30 ml of DCM, followed by the addition of dicyclohexylcarbodiimide (373.45 mg, 1.81 mmol). The mixture was stirred at RT for 15 min, after which PCL (4.80 g, 1.21 mmol) and 4-dimethylaminopyridine (221.12 mg, 1.81 mmol) were added. The reaction was carried out at RT under a N_2_ atmosphere for 48 h and monitored by thin-layer chromatography until completion. The reaction was quenched with 2 ml of deionized water, and the resulting white precipitate was filtered off. Following solvent removal under reduced pressure, the residue was dissolved in 20 ml of ethyl acetate and left to stand at 0 °C for 12 h. The precipitate was collected by filtration. Ethyl acetate was removed via vacuum distillation, and this dissolution–distillation process was repeated 3 times. The final product was dissolved in DCM and slowly added dropwise into precooled, stirred ice-cold ethanol. The precipitate was filtered and vacuum-dried to yield a white PP2000-COOH powder. PP4000-COOH was prepared following the same procedure using COOH-PEG4000-COOH and PCL at a molar ratio of 1:1.

To synthesize PPMal, PP2000-COOH (1 g, 0.16 mmol) and N-(2-hydroxyethyl) maleimide (90 mg, 0.64 mmol) were dissolved in 10 ml of dry DCM. 1-Ethyl-(3-dimethylaminopropyl) carbodiimide hydrochloride (EDC·HCl, 122 mg, 0.64 mmol) and 4-dimethylaminopyridine (1.9 mg, 0.016 mmol) were added, and the reaction was stirred under an N_2_ atmosphere at RT. The reaction mixture was washed sequentially with 0.1 mol/l hydrochloric acid solution and saturated brine. The organic phase was dried over anhydrous Na_2_SO_4_, concentrated, and added dropwise into 50 ml of precooled, stirred ice-cold diethyl ether. The resulting precipitate was filtered and vacuum-dried to obtain white powdered PPMal.

For PPMan synthesis, D-mannosamine·HCl (39 mg, 0.18 mmol) and triethanolamine (22.5 mg, 0.22 mmol) were combined in 1 ml of N,N-dimethylformamide for desalting and dissolution, yielding a D-mannosamine salt solution. PP4000-COOH (1 g, 0.17 mmol) was dissolved in 10 ml of N,N-dimethylformamide, followed by sequential addition of EDC·HCl (59.8 mg, 0.33 mmol) and N-hydroxysuccinimide (38.4 mg, 0.33 mmol). The prepared D-mannosamine salt solution was then added, and the mixture was reacted at RT for 48 h. The resulting solution was transferred into a dialysis bag (molecular weight cutoff [MWCO] = 3,000 kDa) and dialyzed for 48 h to remove unreacted D-mannosamine. The dialysate was freeze-dried (Lgj-12a, Beijing sihuan qihang technology co., ltd., China) to yield PPMan as a white powder.

IVNMs were prepared by the solvent evaporation method [61]. In brief, a total of 10.0 mg of micelle polymers with different ratios of PPMal and PPMan (e.g., PPMal: PPMan = 3:1 by mass, denoted as PPM 3:1) were dissolved in 10 ml of tetrahydrofuran, along with 2.0 mg of Rapa or Nile Red (used for fluorescence labeling in cellular-uptake assays). The solution was added dropwise into 10 ml of deionized water at a rate of 1 drop per second under vigorous stirring (300 rpm). After complete evaporation of tetrahydrofuran, the solution was freeze-dried to obtain nanomicelles, denoted as IVNMs.

For ^1^H NMR analysis, 10 mg of the sample was dissolved in deuterated chloroform (CDCl_3_), with tetramethylsilane used as the internal reference. The spectra were recorded using a 400-MHz ^1^H NMR spectrometer. For FT-IR spectroscopy, 5 mg of the sample was finely ground with an appropriate amount of potassium bromide to form a uniform powder. The mixture was then pressed into a transparent pellet using a pellet press, and the infrared absorption spectrum of the polymer was recorded. For TEM observation, a suspension of IVNMs (1 mg/ml) was prepared and dropped onto a copper TEM grid. After standing for 1 min, excess liquid was removed with filter paper. This process was repeated twice. Subsequently, 1% (w/w) phosphotungstic acid solution was dropped onto the grid for negative staining. After standing for 1 min, the excess stain was blotted off, and the morphology of the IVNMs was observed using TEM.

### Evaluation of Rapa loading capacity, particle characterization, and antigen capture efficiency of IVNMs

The loading capacity of Rapa was determined using ultraviolet-visible spectrophotometry (278 nm, UV2550, SHIMADZU, Japan). A standard calibration curve of Rapa concentration versus absorbance was first established. Freeze-dried samples of IVNMs were resuspended in methanol to release encapsulated Rapa, and the absorbance was measured to calculate the Rapa content within the IVNMs. The particle size and zeta potential of PPM and IVNMs were measured at RT using dynamic light scattering (Nano-Zs90, Malvern, UK) with a 1 mg/ml micelle solution to determine the size distribution and surface charge profile.

The in vitro antigen capture efficacy was evaluated as follows. Log-phase H9c2 cells were seeded in 96-well plates and cultured until approximately 80% confluence. The medium was then removed, and cells were rinsed 2 to 3 times with PBS. Glucose- and serum-free Dulbecco’s modified Eagle’s medium was added, and the plates were placed in a sealed chamber containing a gas mixture of 95% N_2_ and 5% CO_2_, followed by incubation under hypoxic conditions at 37 °C for 12 h. The medium was centrifuged (300 g, 5 min) to remove cellular debris, and the resulting supernatant obtained after oxygen-glucose deprivation treatment was collected as the “antigen pool” for subsequent experiments.

A 1-ml aliquot of each PPM formulation at 1 mg/ml (PPM, PPM (3:1), PPM (2:1), or PPM [1:1]) was mixed with 2 ml of conditioned medium and coincubated at 37 °C and 150 rpm for 24 h. The mixtures were then centrifuged using ultrafiltration tubes (MWCO = 100 kDa) at 8,000 rpm and 4 °C to remove unbound proteins. The resulting antigen-capturing PPM was termed APPM. After centrifugation, the APPM samples were washed twice with PBS, redispersed in PBS, and the filtrates were collected. The antigen capture capacity of PPM was detected using a bicinchoninic acid (BCA) protein quantification kit (20200ES76, Yeasen, China).

Following MI, CM apoptosis increases reactive oxygen species and damage-associated molecular patterns. HMGB1, a representative damage-associated molecular pattern, was used as a marker to assess antigen-capture efficacy. Additionally, LDH, an intracellular enzyme released upon cell membrane damage, was measured as an indicator of cell injury. After coincubation of materials with conditioned medium, the supernatants were collected, and HMGB1 (CSB-E08224r, Cusabio, China) and LDH (JL48282, JONLNBIO, China) levels were quantified using ELISA kits. Comparative analyses of the pre- and postincubation media were performed to evaluate the capture efficiency of PP. For fluorescence imaging, proteins in the antigen pool were labeled with Cyanine5-N-hydroxysuccinimide ester (GEPA15101, Sigma-Aldrich, USA) at 4 °C for 30 min, and PPM micelles were labeled with fluorescein isothiocyanate (FITC; 46950, Sigma-Aldrich, USA). After incubation, fluorescence microscopy was used to visualize and confirm the colocalization and distribution of antigen proteins and PPM micelles.

### Fabrication and characterization of Gel@IVNMs

To synthesize the hydrogel precursor, 10 g of dextran (*Mw* = 8 × 10^5^) was dissolved in 100 ml of deionized water. Sodium periodate (NaIO_4_, 6 g) was dissolved in 60 ml of deionized water, and the NaIO_4_ solution was added dropwise to the dextran solution. The mixture was stirred at RT for 5 h in the dark. Subsequently, 2 ml of ethylene glycol was added to quench the reaction, and the mixture was stirred for an additional hour. The resulting solution was dialyzed against deionized water for 48 h using a dialysis bag (MWCO = 3,500 kDa) and freeze-dried to obtain the product ODex.

The Gel was prepared as follows. A 10% (w/v) ODex solution and a 20% (w/v) gelatin solution were prepared separately in PBS at 37 °C. The ODex and gelatin solutions were then mixed at a 1:1 ratio and incubated at 37 °C to allow gelation. For Gel@IVNMs, the IVNMs were first dispersed in the ODex solution to a final concentration of 400 μg/ml. Upon mixing with the gelatin solution, the final concentration of IVNMs in the hydrogel was 25 μg/ml, and gelation was conducted under the same conditions as described above.

Gel samples were cryofractured in liquid nitrogen and subsequently lyophilized for testing. A portion of the lyophilized sample was sputter-coated with gold (10 nm) and observed using SEM (JSM-7800F Prime, JEOL, Japan) to evaluate morphology.

The Gel was evaluated for gelation time, injectability, and tissue adhesion. Gelation time was tracked by incorporating methyl orange during Gel preparation. Injectability was tested using a 26G needle, followed by application to a knuckle with 10 cycles of flexion to assess tissue adhesion after rinsing with PBS.

Rheological properties were measured using a rheometer (HAAKE RheoWin, Thermo Fisher Scientific, USA). First, Gel discs (30 mm in diameter and 1 mm thick) were prepared using a custom mold. For frequency-sweep tests, strain was fixed at *γ* = 1.0%, the instrument temperature was maintained at 37 °C, and the responses of the Gel’s storage modulus and loss modulus to changes in angular frequency (0.1 to 100 rad/s) were measured. To determine the critical strain at which the Gel network structure is disrupted, frequency was fixed at *f* = 1 Hz, and *G*′ and *G*″ were measured as a function of strain amplitude from 0.1% to 100%.

Swelling and degradation of the injectable Gel were assessed as follows. The Gel was placed in 50-ml centrifuge tubes at RT for 12 h to measure initial swelling. Then, 5 ml of deionized water was added to fully submerge the Gel, and its weight was recorded at 37 °C at multiple time points until equilibrium swelling was achieved and the weights stabilized.

$$\text{Swelling percentage}\left(\%\right)=\frac{\left(W_{q}-W_{p}\right)}{W_{p}}\times 100\%$$ (1)

where *W_p_* is the initial weight of the gel (mg) and *W_q_* is weight of the gel at a specific time point (mg).

For degradation assessment, the Gel was placed in 50-ml centrifuge tubes with 10 ml of PBS (pH = 7.4 and pH = 6.5) and agitated at 37 °C, 50 rpm. The Gel samples were then lyophilized at various time intervals, and their weights were recorded to calculate the degradation percentage.

$$\text{Degradation percentage}\left(\%\right)=\frac{\left(W_{0}-W_{t}\right)}{W_{t}}\times 100\%$$ (2)

where *W*_0_ is the initial weight of the gel (mg) *W_t_* is the weight of the gel at a specific time point (mg).

### Cell isolation and culture

H9c2 cells (American Type Culture Collection, USA) were cultured in high-glucose Dulbecco’s modified Eagle’s medium (PM150210, Procell, China) supplemented with 10% FBS (164210, Procell, China) and 1% penicillin-streptomycin (PB180120, Procell, China) at 37 °C with 5% CO_2_. DC2.4 cells (American Type Culture Collection, USA) were cultured in RPMI-1640 (PM150110, Procell, China) supplemented with 10% FBS (164210, Procell, China) and 1% penicillin-streptomycin (PB180120, Procell, China) under the same conditions.

Bone marrow-derived DCs were isolated from 4-week-old SPF-grade BALB/c mice (Chengdu Dashuo Experimental Animal Co., Ltd., China) that had been fasted for 12 h. The mice were euthanized by cervical dislocation and immersed in 75% ethanol for 5 min. Under sterile conditions, tibias and femurs were isolated, washed, and briefly immersed in 75% ethanol for 30 s. Both ends of the bones were cut open, and precooled RPMI 1640 medium (4 °C) was used with a syringe to flush the bone marrow cavity repeatedly, collecting the rinsing RPMI 1640 medium into a centrifuge tube. The cell suspension was gently pipetted until the cell clusters were fully dispersed, then centrifuged at 1,500 rpm for 5 min. The red blood cells (RBCs) were lysed using RBC lysis buffer for 10 min, followed by washing with PBS.

Cells were resuspended in RPMI 1640 medium containing 20 ng/ml recombinant mouse granulocyte-macrophage colony-stimulating factor (P6006, Beyotime, China) and 20 ng/ml IL-4 (rp154228, Aladdin, China). Ten milliliters of medium was added to each 10-cm culture dish; this was designated as day 0. On day 1, an additional 10 ml of medium was added. On days 2 and 3, half of the medium was replaced. Starting on day 4, the supernatant was collected daily, centrifuged at 1,500 rpm for 5 min to obtain suspension cells, and fresh medium was added. By day 6, all suspension cells were collected as immature DCs. Cells were incubated with CD11c-FITC antibody (E-AB-F0991C, Elabscience, China), and fluorescence images were captured and analyzed using a flow cytometer (NovoCyte, Agilent, USA). For T cell isolation, bone marrow cells were resuspended in PBS containing 2% FBS and 0.5 mM EDTA. Anti-mouse CD3 magnetic beads (11152D, Thermo Fisher Scientific, USA) were added, and the mixture was incubated at 4 °C for 15 to 20 min. The cell suspension was passed through a magnetic separation column and placed in a magnetic field. After removal of the column from the magnetic field, cells were rinsed with buffer to obtain purified CD3^+^ T cells. Rat-derived DCs and primary T cells used for validation experiments were cultured and isolated under the same medium composition, cytokine concentrations, temperature, CO_2_ concentration, medium-change schedule, and stimulation conditions described above, using rat-compatible reagents and antibodies where applicable.

### Biocompatibility and cytotoxicity assays

Biocompatibility and cytotoxicity were evaluated using Calcein-AM/propidium iodide (PI) (C2015S, Beyotime, China) staining and the Cell Counting Kit-8 (CCK-8) assay (40203ES76, Yeasen, China). For live/dead staining, H9c2 cells were seeded in 6-well plates at a density of 2 × 10^5^ cells/well, and DC2.4 cells at 1 × 10^6^ cells/well. After reaching an appropriate confluence (80%), the culture medium was replaced with fresh medium containing nanoparticles at concentrations of 0, 25, 50, 100, or 200 μg/ml, or hydrogel leachates at 0, 25, 50, 100, 200, or 400 μg/ml. Following a 24-h incubation, cells were rinsed 3 times with PBS. A staining solution containing 2 μM Calcein-AM and 4 μM propidium iodide in PBS was then added, and cells were incubated in the dark for 30 min. After washing 2 to 3 times with PBS, fluorescence images were captured using a fluorescence microscope.

For the CCK-8 assay, H9c2 or DC2.4 cells were seeded in 96-well plates at 1 × 10^4^ cells/well and cultured for 24 h. After stabilization, the cells were treated with various concentrations of nanoparticles or hydrogel leachates for 24 h. The absorbance at 450 nm was measured using a microplate reader according to the manufacturer’s instructions, and cell viability (%) was calculated accordingly.

$$\text{Cell viability}\left(\%\right)=\frac{\left(A_{\text{sample}}-A_{\text{blank}}\right)}{\left(A_{\text{control}}-A_{\text{blank}}\right)}\times 100\%$$ (3)

where *A*_sample_ is cells with treatment, *A*_blank_ is cells in the culture medium, and *A*_control_ is cells with equal amount of solvent.

To further evaluate the cytotoxicity of Rapa (R0097, TCI, Japan), H9c2 and DC2.4 cells were coincubated with different concentrations of Rapa for 24 h. The CCK-8 assay was subsequently performed to quantify cell viability, and the absorbance at 450 nm was measured to assess the dose-dependent effects of Rapa on both cell types.

### Evaluation of DC uptake of IVNMs

The uptake of IVNMs by DCs was evaluated as follows. DCs were seeded at a density of 5 × 10^5^ cells per 14-mm confocal dish and incubated overnight for adherence. Free Nile Red (IN0171, Solarbio, China), Nile Red-loaded PP micelles (PP@Nile), Nile Red-loaded PPM micelles (PPM@Nile), or Nile Red-loaded antigen-captured PPM micelles (APPM@Nile) were then added and cocultured with the cells for 4 h. After incubation, the cells were fixed with 2.5% glutaraldehyde for 20 min and rinsed with PBS 2 to 3 times. The nuclei were subsequently stained with Hoechst 33342 (5 μM, B49350, Acmec, China). Cellular uptake was visualized using a confocal laser scanning microscope with randomly selected imaging fields. The proportion of fluorescently positive cells was further quantified by FC using the phycoerythrin (PE) channel.

### Changes in DCs and T-cell phenotypes and cytokine secretion under initial stimulation and restimulation with CLAs

To investigate the effects of CLAs on the phenotypes of DCs and T cells, a coculture system was established using Transwell inserts (pore size: 8 μm). Briefly, 1 × 10^5^ T cells were seeded in the lower compartment, and 5 × 10^4^ immature DCs were placed in the insert. After 24 h of culture to stabilize the cells, the cocultures were divided into 5 groups: (a) Control group, normal culture medium; (b) CLA group, 100-fold diluted CLAs added to the lower compartment; (c) Rapa group, CLAs plus Rapa (1 nmol/l); (d) IVNM group, CLAs plus PPM@Rapa (equivalent to 1 nmol/l Rapa); (d) Gel@IVNMs group, CLAs plus Gel@IVNMs (equivalent to 1 nmol/l Rapa). After 24 h of culture, FC was used to detect DC surface phenotypes, fluorescence microscopy was employed to observe DC morphology, and both FC and immunofluorescence staining were applied to assess T cell phenotypes. For restimulation experiments, groups (b) to (e) were reexposed to CLAs after 7 d of initial culture. Following restimulation, DC phenotypes were again assessed by FC, morphological changes were examined by fluorescence microscopy, and T cell phenotypes were analyzed using FC and immunofluorescence. The supernatants were collected to quantify the cytokines IFN-γ, IL-10, IL-2, and TGF-β (E-EL-M0048, E-EL-M0046, E-EL-M0042, E-EL-0162, all from Elabscience), by ELISA according to the manufacturer’s instructions.

The sequential antibody-labeling protocol for FC was as follows: CD16/32 (E-AB-F0997A, Elabscience, China), Zombie Aqua (423101, BioLegend, USA), CD11c-APC (E-AB-F0991E, Elabscience, China), CD80-FITC (E-AB-F0992C, Elabscience, China), and PD-L1-PE (E-AB-F1132D, Elabscience, China), with cell labeling performed according to the instructions. The sequential labeling protocol for T cell phenotype detection by FC was: CD16/32 (E-AB-F0997A, Elabscience, China), Zombie Aqua (423101, BioLegend, USA), CD4-APC (E-AB-F1097E, Elabscience, China), CD25-PE (E-AB-F1102D, Elabscience, China), and CD127-FITC (E-AB-F1023C, Elabscience, China). ELISA was performed according to the supplied instructions. For T cell immunofluorescence labeling, nuclei were labeled with 4′,6-diamidino-2-phenylindole (DAPI) (E-CK-A163, Elabscience, China), CD4 with FITC (E-AB-F1097C, Elabscience, China), and CD127 with Alexa Fluor 555 (16-1271-85, eBioscience, USA). Additionally, DCs were cultured independently following the same stimulation conditions, and cytokine concentrations were quantified by ELISA. Unless otherwise indicated, products listed in this paragraph were obtained from Elabscience (China).

After cell collection, cell pellets were lysed in radioimmunoprecipitation assay buffer (P0013B, Beyotime, China) supplemented with protease and phosphatase inhibitors. Lysates were incubated on ice for 30 min and centrifuged at 4 °C. Protein concentrations were determined using a BCA assay (P0010, Beyotime, China). Equal amounts of protein (20 to 30 μg) were separated by sodium dodecyl sulfate-polyacrylamide gel electrophoresis (P0012AC, Beyotime, China) and transferred onto polyvinylidene fluoride membranes (FFP70, Beyotime, China). Membranes were blocked with 5% nonfat milk at RT for 1 h, followed by overnight incubation at 4 °C with primary antibodies against p-mTOR (Ser2448; AF3308, Affinity Biosciences, USA), mTOR (AF6309, Affinity Biosciences, USA), p-p70S6K (Thr389; AF3228, Affinity Biosciences, USA), p70S6K (9202, CST, USA), p-S6 (Ser235/236; 4856S, CST, USA), and S6 (ab127980, Abcam, UK). After washing with tris-buffered saline with Tween 20, membranes were incubated with horseradish peroxidase-conjugated secondary antibody (7074, CST, USA) for 1 h at RT. Protein bands were visualized using enhanced chemiluminescence and captured using an imaging system. Band intensities were analyzed using ImageJ software (v1.54, NIH, USA), and relative protein expression was expressed as the ratio of phosphorylated protein to corresponding total protein.

### Animals

Male Sprague-Dawley (SD) rats (6 to 8 weeks old, 200 ± 25 g) were purchased from Chengdu Dossy Experimental Animals CO., LTD. All animal procedures were approved by the Medical Ethics Committee of The Third People’s Hospital of Chengdu (CDTPH2025021601). Animals were housed under controlled conditions (12-h light/dark cycle; ambient temperature of 25 ± 1 °C; humidity of 60% ± 5%) in a well-ventilated, noise-free environment with ad libitum access to food and water. Rats were fasted for 12 h prior to surgery, with free access to water. Short-term experiment: Experimental rats were randomly divided into 5 groups (*n* = 8): (a) Sham operation group (Control group); (b) MI group; (c) Gel@PPM group; (d) Gel@PP@Rapa group; (e) Gel@IVNMs group. For the treatment groups, following coronary ligation, the pericardium was gently lifted, and an intramyocardial injection was performed at a total volume of 100 μl/kg body weight. The procedure was conducted at 37 °C to prevent hypothermia. After suturing the thoracic cavity, penicillin was administered intramuscularly at 250 mg/kg per rat for 3 consecutive days. All relevant physiological and biochemical parameters were then evaluated.

For the long-term experiment, rats were randomly assigned to 5 groups (*n* = 8 per group): Control, Gel@PPM, Gel@PP@Rapa, Gel@OVA-IVNMs, and Gel@IVNMs. Control rats underwent thoracotomy without effective coronary artery ligation. The Gel@PPM group underwent MI induction and received drug-free Gel@PPM. After MI induction, the Gel@PP@Rapa, Gel@OVA-IVNMs, and Gel@IVNMs groups received the corresponding treatment formulations. At each long-term time point, 3 animals per group were randomly selected for downstream analyses.

The long-term experiment used the same formulation doses as the short-term experiment. Immediately after coronary artery ligation, all treatment formulations were delivered by intramyocardial injection into the infarct region at a total volume of 100 μl/kg body weight per rat. Gel@IVNMs contained 25 μg/ml IVNMs, and Gel@PPM and Gel@PP@Rapa were prepared at the same material and Rapa concentrations used in the short-term experiment. Gel@OVA-IVNMs was prepared by immersing preformed Gel@IVNMs in an OVA solution (100 μg/ml) for 12 h before administration. The Control group received an equal volume of vehicle.

On days 7 and 56 after MI, 3 animals per group were randomly selected, anesthetized, and euthanized by cardiac perfusion. The anterior mediastinal lymph nodes and cardiac tissues were immediately collected from these animals. The lymph nodes were used for FC and ex vivo antigen-restimulation experiments, whereas the cardiac tissues were allocated to immunofluorescence staining and cytokine quantification according to the downstream analysis.

### MI model

The MI model was established by surgical ligation of the LAD coronary artery in SD rats. Briefly, rats were anesthetized and fixed in a supine position on a heating pad to maintain body temperature. The surgical areas of the neck and left chest were depilated and disinfected with iodophor. A midline incision was made in the neck, and the muscles were bluntly dissected to expose the trachea. An 18G tracheal catheter was inserted and connected to a small animal ventilator to ensure regular respiration. A left thoracotomy (~4 cm) was performed approximately 3 mm lateral to the left sternal border. The subcutaneous tissue, pectoralis major, and serratus anterior were bluntly separated to expose the ribs. The third intercostal muscle was carefully incised, and the pleura was punctured during exhalation to enter the thoracic cavity. The ribs were retracted, and the pericardium was gently torn to expose the heart. A sterile cotton pad was placed under the left lung for isolation. The left atrial appendage was identified, and the LAD coronary artery was ligated approximately 2 mm below the junction of the pulmonary artery cone and the left atrial appendage using an 8-0 suture. Successful ligation was confirmed by immediate pallor of the myocardium distal to the ligation site and enlargement of the left atrial appendage. The procedure was performed rapidly and accurately to minimize trauma to the rats.

After confirming hemostasis, the thoracic cavity was gradually closed, taking care not to suture the heart, lungs, or blood vessels. Residual air and fluid were removed using a syringe before the final closure. The surgical site was disinfected, and after recovery from anesthesia, the tracheal tube was removed, and airway secretions were cleared. In the sham operation group, the same surgical procedures were performed without ligation.

### Biosafety evaluation

Biosafety was assessed through hematological analysis and histopathological examination of major organs. Briefly, rats were euthanized, and blood samples were collected from the abdominal aorta for detection of routine hematological and serum biochemical indices. Major organs, including the liver, spleen, lung, and kidney, were collected and fixed in 4% paraformaldehyde solution for 24 h. The fixed tissues were processed, embedded in paraffin, sectioned, and stained with H&E. Histopathological changes were examined and imaged using a digital slide scanner to evaluate potential tissue toxicity.

Hemocompatibility was evaluated following a standard hemolysis test. Whole blood was collected from normal SD rats using sodium citrate–coated tubes. A 1-ml aliquot of blood was transferred to a 5-ml centrifuge tube, and physiological saline was added to bring the total volume to 4 ml. The mixture was centrifuged at 2,500 rpm for 10 min, the supernatant was removed, and the precipitate was repeatedly washed with physiological saline until the supernatant became clear. The RBCs obtained after washing and centrifugation were resuspended in physiological saline to prepare a 4% RBC stock solution, which was stored at 4 °C until use. Preprepared hydrogel leachate (200 μg/ml) was coincubated with 500 μl of RBCs, with attention to adding the material first, then slowly adding the RBCs. Pure water and physiological saline served as the positive and negative controls, respectively. The mixture was incubated on a shaker at 37 °C for 1 h, then centrifuged at 3,000 rpm for 10 min. The supernatant was collected to measure absorbance at 540 nm, and the hemolysis rate was calculated.

$$\begin{array}{c} \text{Hemolysis rate}\left(\%\right)=\\ \frac{\left({\mathrm{OD}}_{\text{simple}}-{\mathrm{OD}}_{\text{Negative control}}\right)}{\left({\mathrm{OD}}_{\text{Positive control}}-{\mathrm{OD}}_{\text{Negative control}}\right)}\times 100\% \end{array}$$ (4)

where *OD*_sample_ is the optical density of the test-mixture supernatant, *OD*_negative control_ is the optical density of the normal-saline supernatant, and *OD*_positive control_ is the optical density of the pure-water supernatant.

### Therapeutic effects of Gel@IVNMs in a rat MI model

Cardiac tissue morphology was evaluated by H&E staining (60524ES60, Yeasen) and Masson’s trichrome staining (60532ES66, Yeasen). Rats were anesthetized and euthanized, and the thoracic cavity was opened. While the heart was still beating, a perfusion needle was inserted into the left ventricle with an insertion depth of approximately 3 to 4 mm. Care was taken to avoid tissue injury. The right atrial appendage was incised, and perfusion was performed with PBS. The temperature and pressure of the perfusate were kept stable to ensure perfusion efficiency until the forelimbs and lungs of the rats turned white. Subsequently, 4% paraformaldehyde (60536ES76, Yeasen) was used for fixation until severe twitching of the rat forelimbs was observed, with the forelimbs and neck showing stiffness. After completion of perfusion, the heart or other relevant tissues were removed as required by the experiment, placed in 4% paraformaldehyde fixative, and stored at 4 °C. Next, myocardial tissue was processed through procedures such as dehydration, clearing, paraffin infiltration, embedding, and sectioning to prepare 4-μm-thick tissue sections. The sections were then subjected to H&E and Masson trichrome staining, and images were captured using a digital slide scanner for further analysis of structural changes in cardiac tissue.

### Preparation of single-cell suspensions from cardiac-draining lymph nodes and analysis of DC phenotypes

Immediately after euthanasia, the anterior mediastinal lymph nodes were aseptically excised and placed in ice-cold fluorescence-activated cell sorting buffer (PBS containing 2% FBS and 2 to 5 mM EDTA). After surrounding fat and connective tissue were removed, the lymph nodes were mechanically dissociated in a sterile culture dish and filtered through a 70-μm cell strainer to obtain a uniform single-cell suspension. The cells were washed with PBS, centrifuged, resuspended, and counted using a hemocytometer.

To evaluate lymph node DC phenotypes, equal numbers of cells were subjected to flow-cytometric staining. Dead cells were first excluded using a viability reagent (E-CK-A169, Elabscience, China). Cells were then surface-stained with fluorophore-conjugated anti-rat CD11b/c (AN00651E, Elabscience, China), MHC-II (AN00653D, Elabscience, China), and PD-L1 (E-AB-F1132UC, Elabscience, China) antibodies. After incubation for 20 min at RT in the dark, the cells were washed, resuspended in flow cytometry buffer, and analyzed using a flow cytometer (NovoCyte, Agilent, USA).

Flow cytometry data were analyzed using NovoExpress v1.6.1. Cell debris was excluded on the basis of forward-scatter and side-scatter characteristics, followed by doublet exclusion using parameters including FSC-A and FSC-H. CD11b/c and MHC-II expression was then assessed within the viable singlet population. CD11b/c^+^MHC-II^+^ cells were used as the principal population for DC phenotype analysis, and PD-L1 expression within this population was quantified to evaluate an immunoregulatory/tolerance-associated phenotype. Gates were defined using unstained controls.

### Ex vivo antigen stimulation of lymph node cells and Treg analysis

To evaluate the effects of the different treatments on antigen-associated regulatory T cell responses, the lymph node single-cell suspensions described above were seeded in 24-well plates at 5 × 10^5^ cells/well. After culture for 24 h at 37 °C in 5% CO_2_, adherent cells were removed and the suspended-cell fraction was collected for the subsequent stimulation experiments.

The suspended cells from each sample were divided equally among 3 stimulation conditions: (a) an unstimulated condition, in which only standard culture medium was added; (b) an OVA condition, in which OVA (Elabscience, E-PP-1669, China) was added to a final concentration of 100 μg/ml; and (c) a CLA condition, in which CLAs were added to a final total-protein concentration of 100 μg/ml. The CLAs were prepared from autologous cardiac tissue obtained from the same animal as the corresponding lymph node sample; thus, lymph node cells from each rat were stimulated with CLAs derived from that rat. To prepare the CLAs, rat ventricular myocardium was minced and homogenized in ice-cold lysis buffer at approximately 1:3 (w/v) (100 mM tris, pH 7.4, 150 mM NaCl, 1 mM EGTA, 1 mM EDTA, 1% Triton X-100, and 0.5% sodium deoxycholate) supplemented immediately before use with protease and phosphatase inhibitor cocktails. The homogenate was centrifuged at 8,000 × g for 15 min, the supernatant was collected, and the total protein concentration was determined using a BCA assay.

Tregs were quantified by FC. Live and dead cells were first discriminated using a viability reagent (E-CK-A169, Elabscience, China), followed by Fc-receptor blocking (E-AB-F0997A, Elabscience, China). Cells were then surface-stained with fluorophore-conjugated anti-rat CD4 (E-AB-F1105C, Elabscience, China) and CD25 (PE-30314, Finetest, China) antibodies. After surface staining, cells were fixed and permeabilized with Fixation/Permeabilization Concentrate and Permeabilization Buffer (E-CK-A108, Elabscience, China) according to the manufacturer’s instructions and intracellularly stained with an anti-rat FOXP3 antibody (E-AB-F1351J, Elabscience, China).

Data were acquired using a NovoCyte flow cytometer. After sequential gating of the cellular, singlet, and viable-cell populations, CD4^+^ T cells were identified, and CD25 and FOXP3 coexpression was assessed within the CD4^+^ population. CD4^+^CD25^+^FOXP3^+^ cells were defined as Tregs. The principal outcome was the proportion of Tregs among all suspended cells across the different in vivo treatment groups, time points, and ex vivo antigen-stimulation conditions. Positivity thresholds for FOXP3 and CD25 were defined using negative staining controls.

### Multiplex immunofluorescence staining of myocardial tissue

To evaluate inflammatory-cell infiltration and macrophage phenotypes in the myocardium after MI, cardiac tissues were collected on days 7 and 56. Samples from the infarct and peri-infarct regions were fixed in 4% paraformaldehyde (60536ES76, Yeasen, China), embedded in paraffin, and sectioned at a thickness of 4 μm.

After deparaffinization and rehydration, sections were blocked with 5% normal goat serum for 30 min at 37 °C and then incubated with primary antibodies against CD68, iNOS, and CD206. The antibodies were Alexa Fluor 647-conjugated anti-CD68 (ab305214, Abcam, China), Alexa Fluor 455-conjugated anti-iNOS (ab209594, Abcam, China), and Alexa Fluor 488-conjugated anti-CD206 (ab318602, Abcam, China).

Images were acquired using an Axio Observer fluorescence microscope (Axio Observer, Carl Zeiss, Germany). Laser power, exposure time, gain, and all other acquisition parameters were kept constant across experimental groups. For each animal, 3 nonoverlapping fields were randomly selected within infiltrated regions and analyzed using ImageJ software (v1.54, NIH, USA).

### ELISA measurement of cytokines in myocardial tissue

To evaluate the effects of the different treatments on the local inflammatory and immunoregulatory microenvironment after MI, tissue from the infarct region was collected on days 7 and 56. A defined mass of myocardial tissue was homogenized in 150 to 250 μl of ice-cold radioimmunoprecipitation assay lysis buffer per 20 mg of tissue. Homogenates were centrifuged at 12,000 rpm for 15 min at 4 °C, and the supernatants were collected for subsequent analyses.

The total protein concentration in each tissue lysate was measured using a BCA protein assay kit (P0010, Beyotime, China), and samples were normalized to total protein content. Rat IL-2 (MM-0701M1, MEIMIAN, China), IFN-γ (MM-052002, MEIMIAN, China), TGF-β (MM-20594R1, MEIMIAN, China), and IL-10 (MM-0195R1, MEIMIAN, China) were then quantified using ELISA kits.

Absorbance at 450 nm was measured using a microplate reader (SPECTROstar Nano, BMG LABTECH, Germany), and IL-2, IFN-γ, TGF-β, and IL-10 concentrations were calculated from the corresponding standard curves.

The effects of Gel@IVNMs on apoptosis and angiogenesis were evaluated as follows. After dewaxing, rehydration, and antigen retrieval of cardiac paraffin sections, the sections were covered with 5% normal goat serum and blocked at 37 °C for 30 min. According to the instructions, the primary antibodies against CD31 (ab28364, Abcam, UK) and α-SMA (MAB1420, Bio-Techne, USA) were diluted as required, the blocking solution was removed, and the primary antibody working solution was added dropwise. The sections were placed flat in a humidified chamber and incubated overnight at 4 °C. After rewarming at RT for 15 min, the primary antibody working solution was removed, and the sections were rinsed 3 times with PBS. Secondary-antibody solutions—Alexa Fluor 488 (A-21206, Thermo Fisher Scientific, USA) for CD31 and Alexa Fluor 594 (A-11032, Thermo Fisher Scientific, USA) for α-SMA—were prepared according to the supplied instructions, added to the sections, and incubated for 1 h at RT in the dark. The secondary antibody working solution was then removed, and the sections were rinsed 3 times with PBS. DAPI (2 μM) working solution was incubated with the sections at RT in the dark for 15 min, after which the DAPI working solution was removed. After 3 washes with PBS, the sections were mounted, fluorescence images were acquired, and fluorescence intensity was quantified. Similarly, paraffin sections were stained using the TUNEL apoptosis kit (40306ES60, Yeasen, China) according to the supplied instructions, fluorescence images were acquired, and fluorescence intensity was quantified.

Cardiac function was assessed by echocardiography (Vevo 3100, FUJIFILM VisualSonics, Canada). Rats were anesthetized in an induction chamber and then positioned supine on an imaging platform. Rats were continuously anesthetized with isoflurane at a concentration of 1% to 2% and a flow rate of 0.6 to 1 l/min via an anesthesia mask. During this process, conductive gel was applied to the electrode pads, and the limbs of the rats were fixed with tape to ensure accurate collection of electrocardiogram signals. Body temperature and respiration were continuously monitored throughout imaging. M-mode echocardiographic data were acquired using a small-animal ultrasound imaging system, and cardiac functional parameters were analyzed.

Statistical analysis: Data were analyzed and processed using GraphPad Prism software (version 8, GraphPad Software, USA), and statistical significance was determined by 1-way analysis of variance.

## Data Availability

The data are freely available upon request.
