# Supplementary material for: Inverse Vaccine Nanomimics: In situ Antigen-Specific Immune Tolerance Induction for Durable Cardiac Repair Post-Myocardial Infarction
Source: Research (Wash D C). 2026 Oct 21;9:1464. doi: 10.34133/research.1464 (PMC13683952; doi:10.34133/research.1464)
Supplement: Supplementary file 1 [file research.1464.f1.pdf]

Supporting Information

Inverse Vaccine Nano-Mimics: *In Situ* Antigen-Specific Immune Tolerance Induction for Durable Cardiac Repair Post Myocardial Infarction

Tian Yue, Jiali Yang, Qing Ma, Jian He, Ying Wang, Zhilu Yang* and Jun Hou**

*Corresponding Authors:

e-mail: wangying277@outlook.com; zhiluyang1029@smu.edu.cn; houjun@swjtu.edu.cn.

The PDF file includes:
Experimental Section
Figs. S1 to S9
Tables S1 to S4

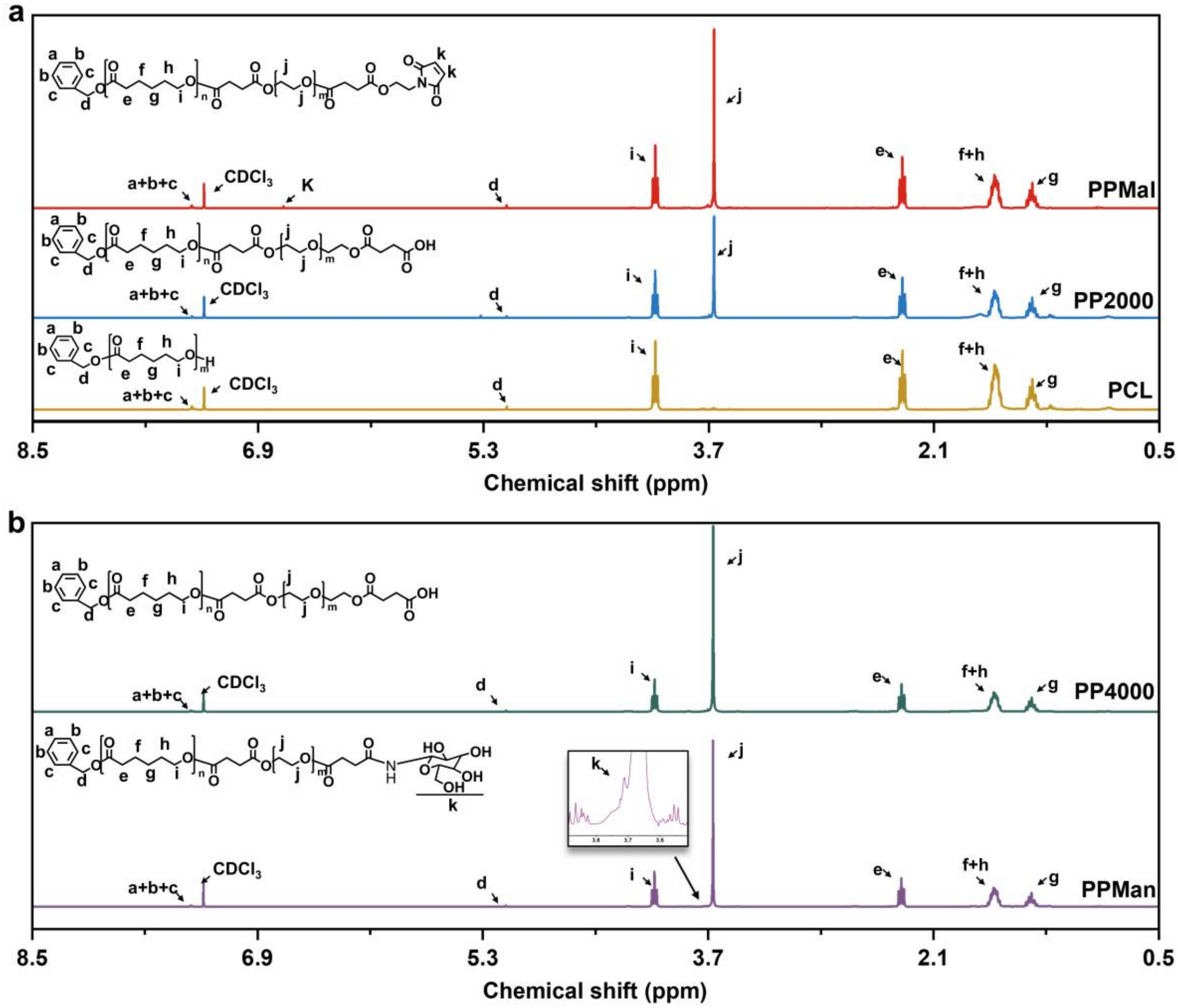

Supplementary Fig. S1. Identification of micelle polymer structures. a, ^{1}H NMR spectra of PCL, PP2000, and PPMal; b, ^{1}H NMR spectra of PP4000 and PPMan.

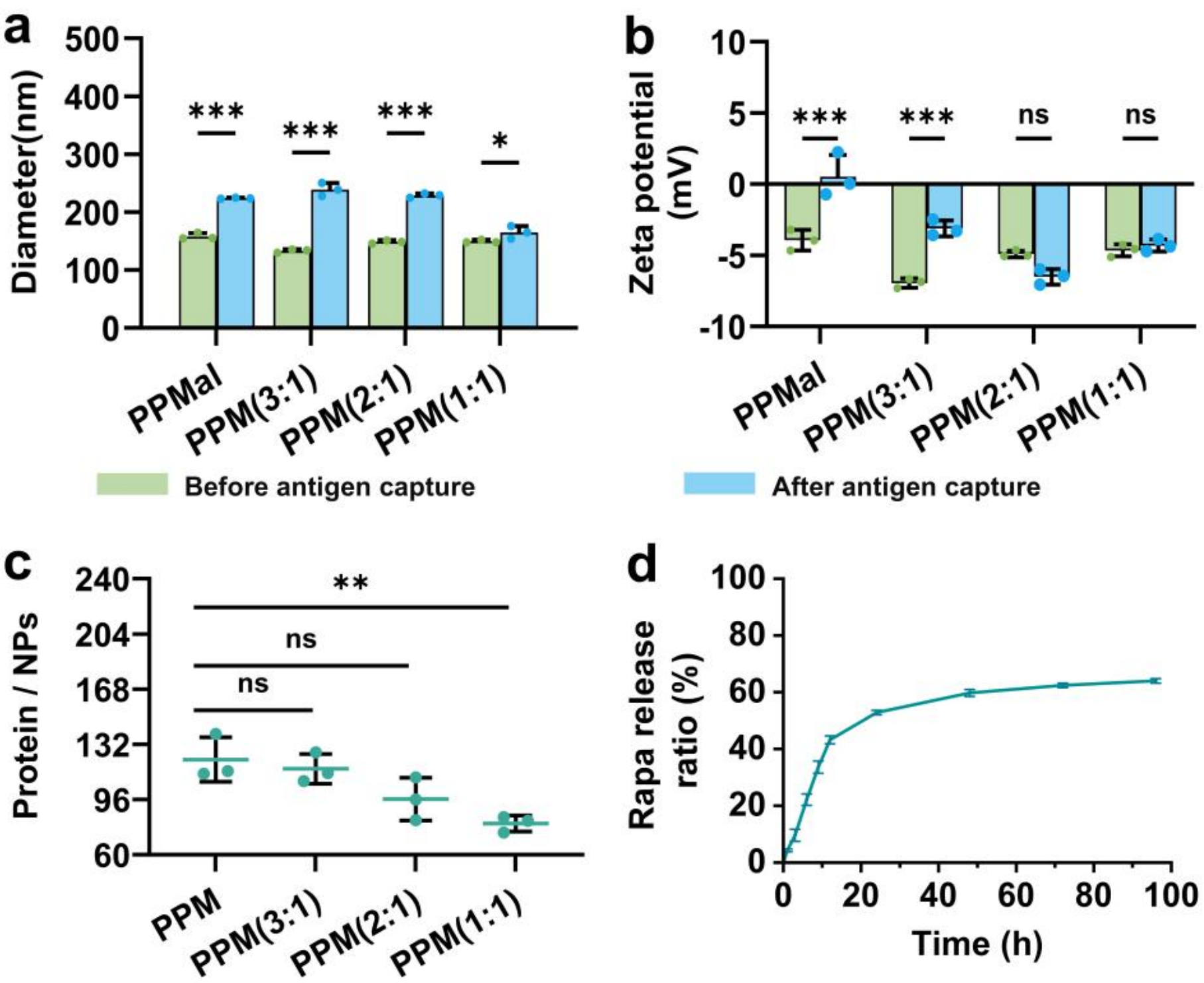

Supplementary Fig. S2. Nanoparticle screening based on antigen-capture capacity. a, Changes in particle size before and after protein adsorption for PPMal and PPMal:PPMan ratios of 3:1, 2:1, and 1:1; b, Changes in zeta potential before and after protein adsorption for PPMal and PPMal:PPMan ratios of 3:1, 2:1, and 1:1; c, Protein adsorption amounts for nanoparticles with PPMal:PPMan ratios of 3:1, 2:1, and 1:1; d, Rapa release from PPM over time. Data are presented as mean ± SD, with individual points representing biological replicates where applicable. Statistical significance is indicated as ns, not significant; *P < 0.05; **P < 0.01; ***P < 0.001; ****P < 0.0001.

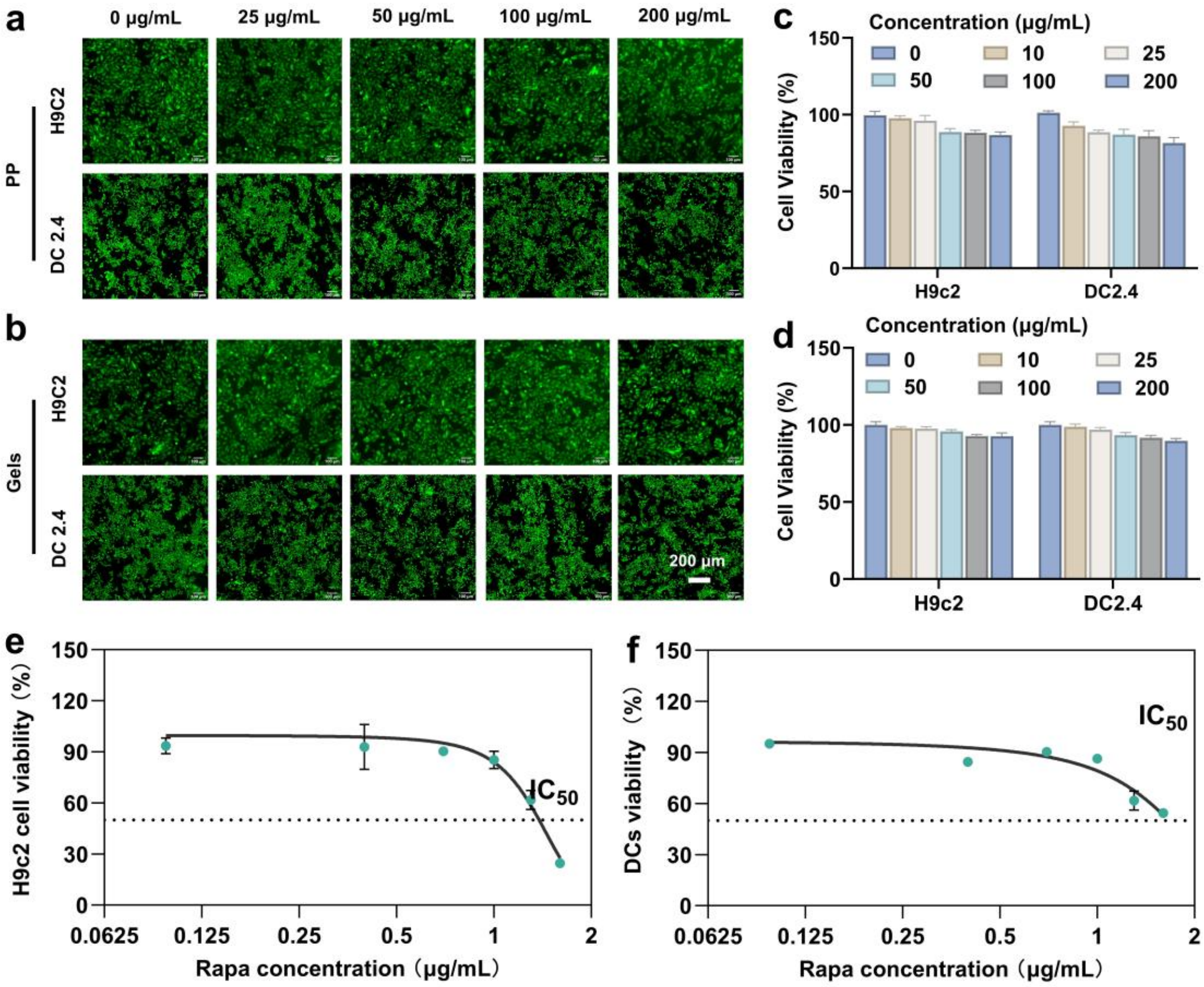

Supplementary Fig. S3. Biocompatibility of IVNMs and Gel, and cytotoxicity of Rapa. a-b, Cytotoxicity assay results for H9c2 and DC2.4 cells treated with IVNMs and Gel, with green indicating live cells and red indicating dead cells; c-d, Quantification of cytotoxicity for H9c2 and DC2.4 cells treated with IVNMs and Gel; e-f, Cytotoxicity and IC_{50} curves of Rapa in H9c2 and DC2.4 cells. Data are presented as mean ± SD, with individual points representing biological replicates where applicable. Statistical significance is indicated as ns, not significant; *P < 0.05; **P < 0.01; ***P < 0.001; ****P < 0.0001.

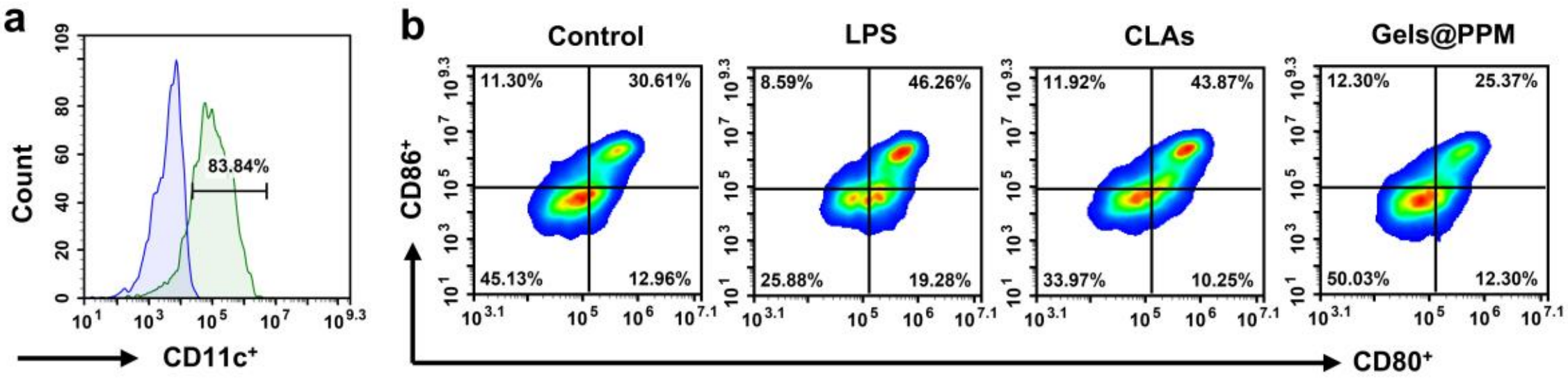

Supplementary Fig. S4. DC isolation and maturation. a, Phenotypic identification of isolated DCs, with positive cells identified by $CD11c^+$ expression; b, Flow-cytometry results showing the effects of LPS, CLAs, and Gel@PPM on DC maturation. Data are presented as mean ± SD, with individual points representing biological replicates where applicable. Statistical significance is indicated as ns, not significant; *P < 0.05; **P < 0.01; ***P < 0.001; ****P < 0.0001.

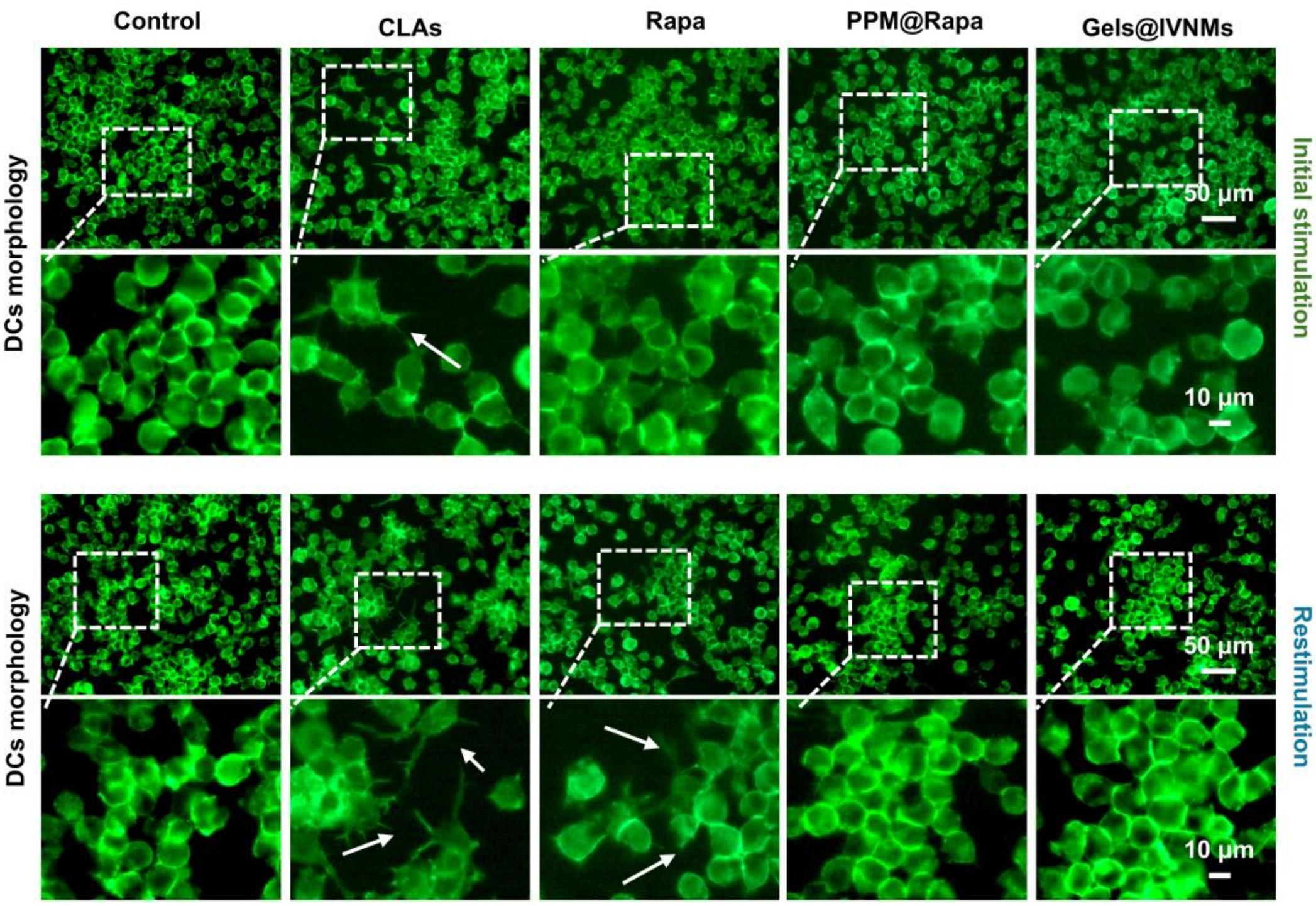

Supplementary Fig. S5. Morphological changes in DCs. Fluorescence images of DC morphology after cytoskeleton staining; blue indicates cell nuclei and green indicates the cytoskeleton.

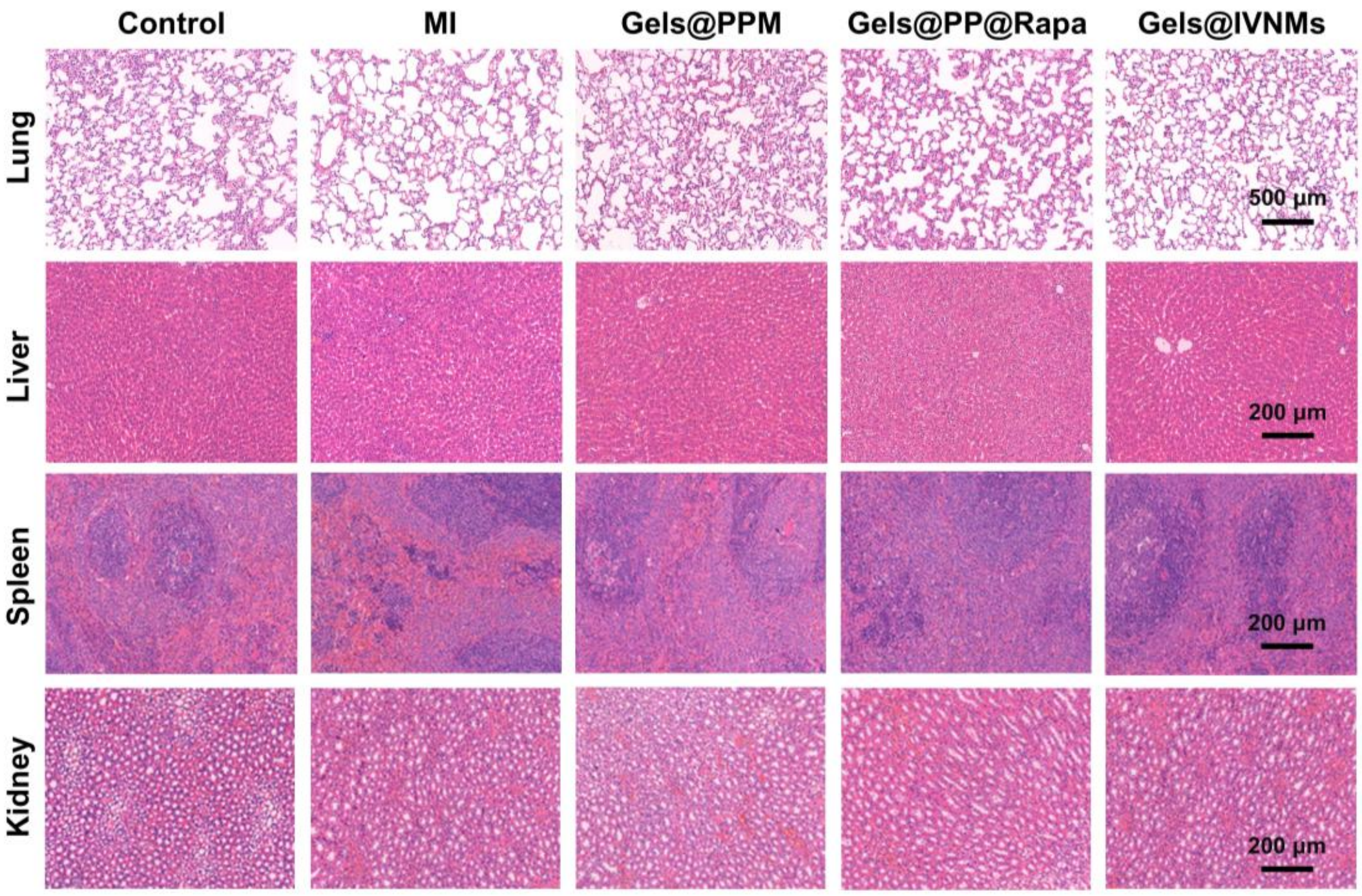

Supplementary Fig. S6. Biological safety. H&E staining of major organs.

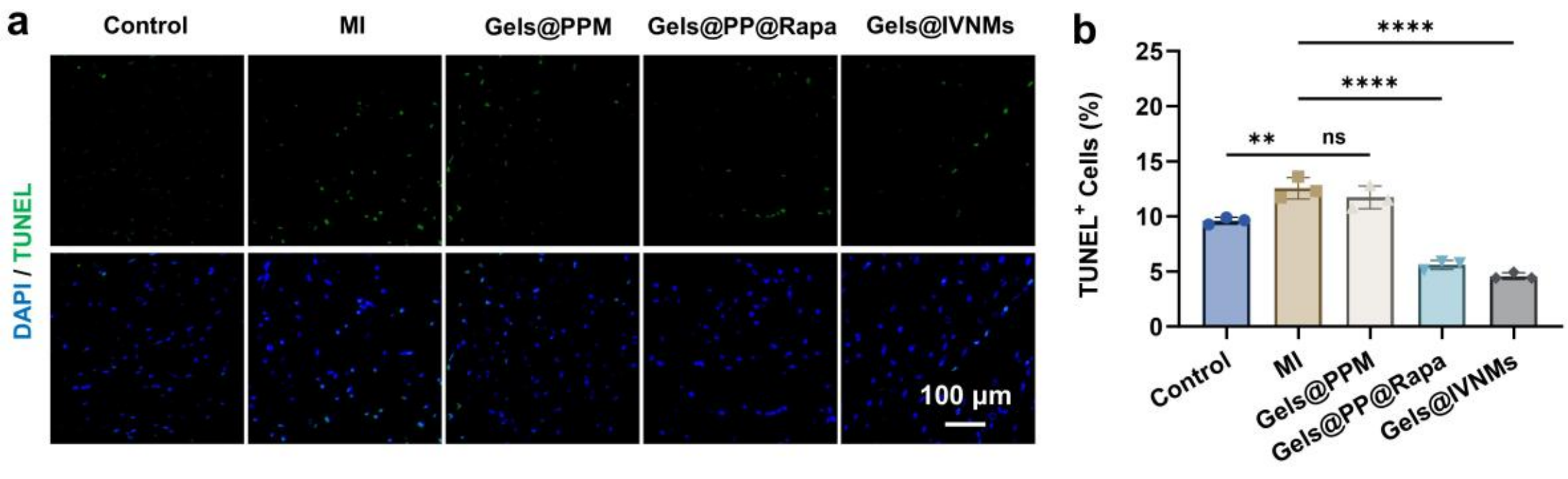

Supplementary Fig. S7. Cardiomyocyte apoptosis. a, TUNEL staining of cardiac sections, with green indicating apoptotic cells and blue indicating cell nuclei; b, Quantification of apoptotic cells.

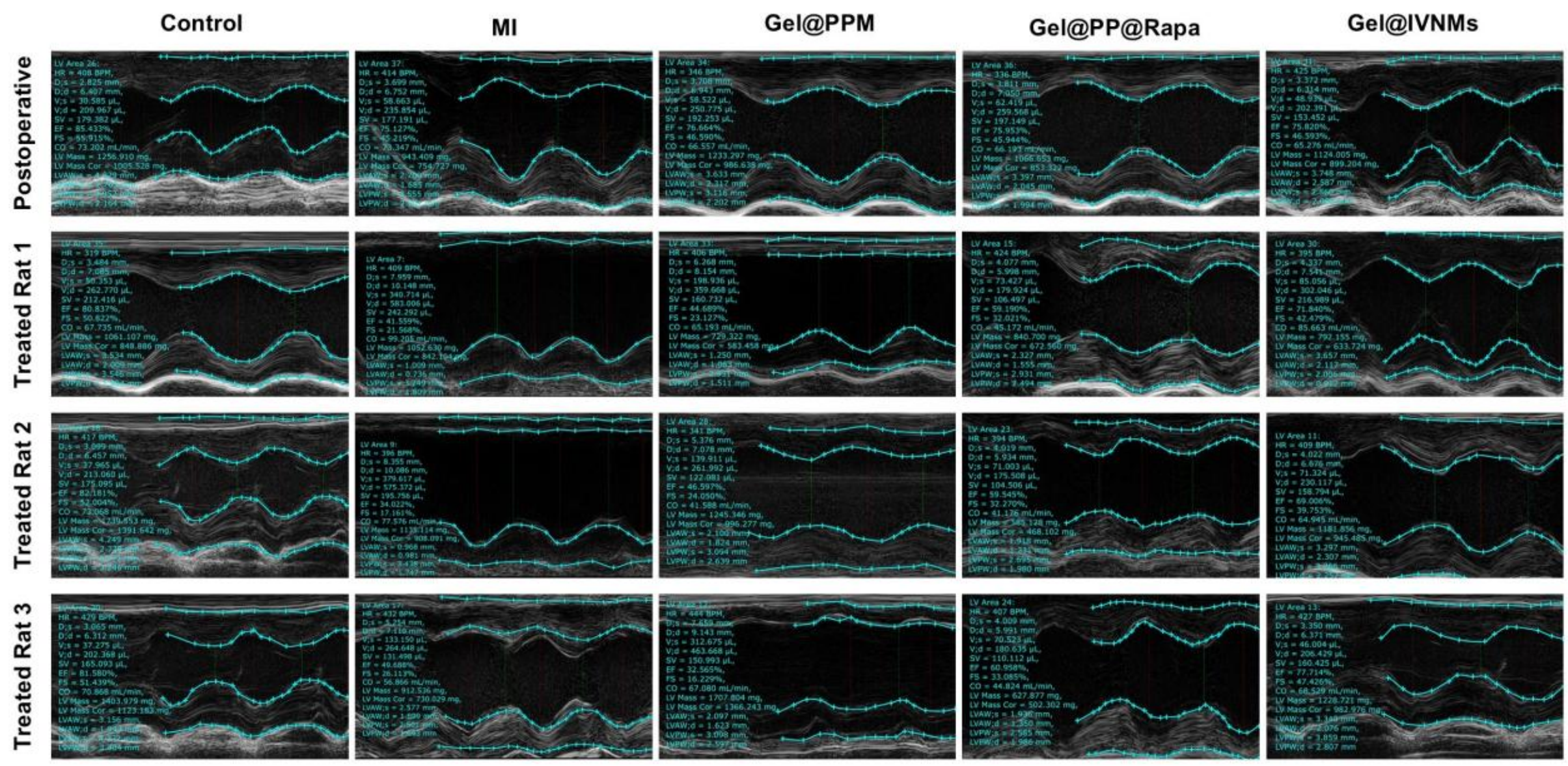

Supplementary Fig. S8. Echocardiographic assessment of cardiac function in rats following establishment of the MI model and after treatment, with results shown for each individual rat.

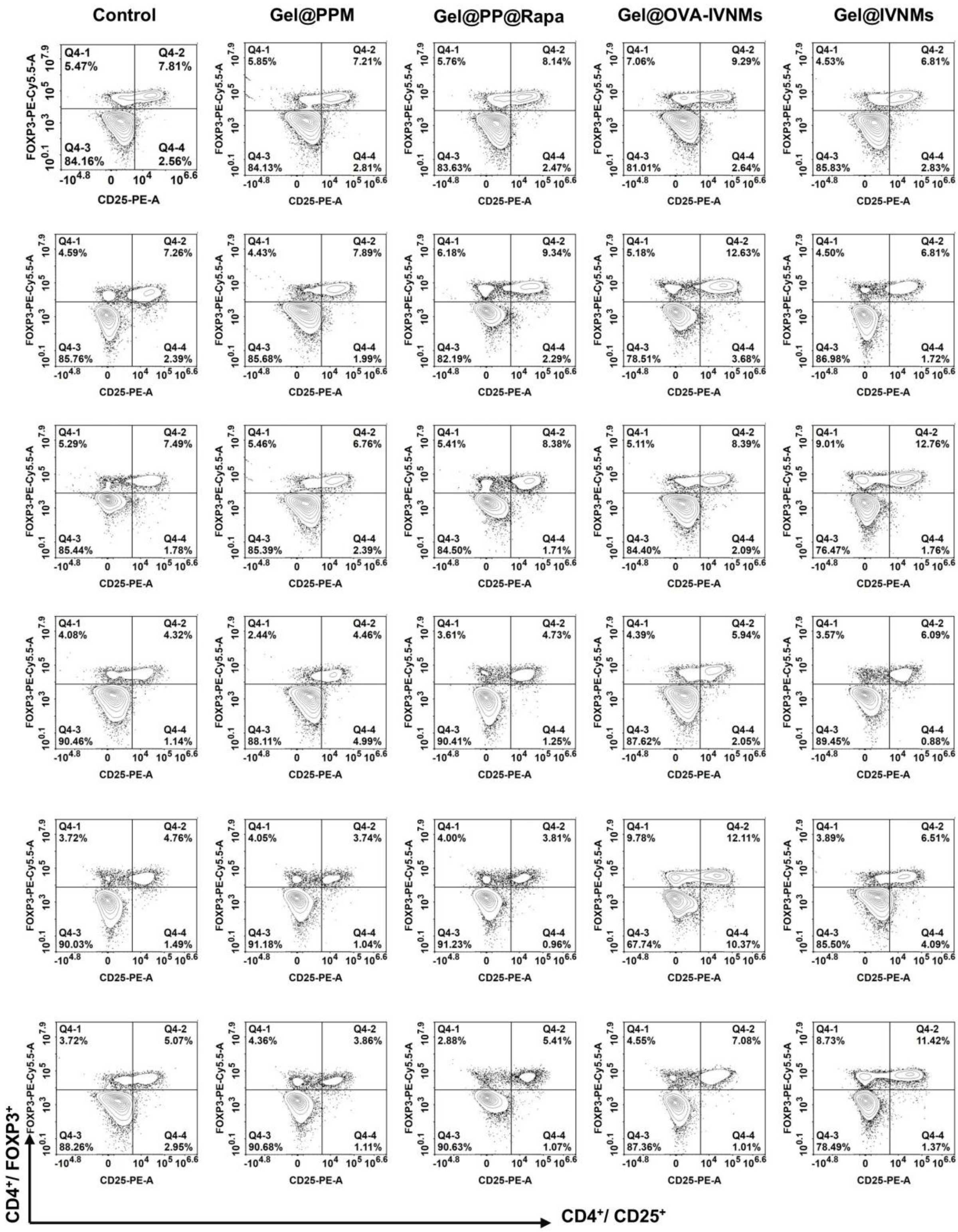

Supplementary Fig. S9. Detailed flow-cytometry plots for lymph-node cells from each treatment group at days 7 and 56 following stimulation with OVA or autologous CLAs from the same rat.

Supplementary Table S1. Drug loading and encapsulation efficiencies of PPM@Rapa.

Rapa/PPM (mg/mg)	LC (%)	EE (%)
1:10	4.14 ± 0.16	45.43 ± 0.59
2:10	18.07 ± 0.07	63.24 ± 0.23
3:10	12.29 ± 0.68	53.26 ± 0.28

Supplementary Table S2. Hematological data from rats.

Parameters	Full name	Result 1	Result 2	Result 3	Unit	Reference value
Wbc	White blood cell count	6.17	14.32	2.14	10^9/L	1.90 - 16.80
Neu#	Neutrophil count	1.74	3.16	0.63	10^9/L	0.35 - 6.30
Lym#	Lymphocyte count	3.84	10.09	1.24	10^9/L	0.91 - 12.20
Mon#	Monocyte count	0.55	1.03	0.26	10^9/L	0.08 - 2.30
Eos#	Eosinophil count	0.04	0.04	0.01	10^9/L	0.00 - 1.01
Bas#	Basophil count	0	0	0	10^9/L	0.00 - 0.20
Neu%	Neutrophil percentage	28.1	22.1	29.3	%	7.3 - 50.0
Lym%	Percentage of lymphocytes	62.3	70.5	57.7	%	40.0 - 88.9
Mon%	Monocyte percentage	8.9	7.2	12.5	%	2.0 - 18.0
Eos%	Eosinophil percentage	0.7	0.2	0.5	%	0.0 - 7.0
Bas%	Percentage of basophils	0	0	0	%	0.0 - 1.5
Rbc	Red blood cell count	6.75	8.37	4.06	10^12/l	5.00 - 9.80
Hgb	Hemoglobin	144	164	83	g/L	110 - 170
Hct	Hematocrit	39.6	44.1	23.3	%	32.0 - 53.0
Mcv	Mean corpuscular volume	58.7	52.7	57.3	fL	50.0 - 67.0
Mch	Mean corpuscular hemoglobin concentration	21.3	19.6	20.5	pg	16.0 - 23.0
Mchc	Mean corpuscular hemoglobin concentration	364	371	357	g/L	310 - 370

Supplementary Table S3. Main reagents, specifications, manufacturers, and abbreviations.

Unless otherwise specified, fluorescent dyes and detection kits were purchased from Beyotime Biotechnology Co., Ltd., China. ELISA kits were purchased from Jiangsu Meimian Biotechnology Co., Ltd., China. Fluorescent antibodies were purchased from eBioscience, USA. Chemical reagents were purchased from Adamas, China.

**Materials**	**Specifications**	**Manufacturer**	**Abbreviation**
polyethylene glycol	Mn = 2000, AR<br>Mn = 4000, AR	TCI, Japan	PEG
stannous chloride	99%	Macklin, China	——
Dicyclohexylcarbodiimide	99%	TCI, Japan	DCC
dextran	Mn = 7w	Xiya, China	Dex
sodium periodate	99%+	Macklin, China	——
Type A gelatin	BR	Yuanye, China	Gelatin，GEL
PBS buffer	500 mL	Procell, China	——
RPMI-1640 medium	500 mL	Gibco, USA	
DMEM medium	500 mL	Servicebio, USA	
fetal bovine serum	500 mL	Gibco, USA	FBS
penicillin-streptomycin	100 mL	Wuhan Procell Life Technology Co., Ltd., China	
Recombinant Mouse IL-4	10 μg	Suzhou Nearshore Protein Technology Co., Ltd., China	
Recombinant Mouse GM-CSF	10 μg	Suzhou Nearshore Protein Technology Co., Ltd., China	

lipopolysaccharide	10 mg	Sigma, USA	LPS

Supplementary Table S4. Main instruments, models, manufacturers, and countries.

Laboratory equipment	Model	Manufacturer
Freeze dryer	LGJ-12A	Beijing Sihuan Qihang Technology Co., Ltd., China
Dynamic light scattering instrument (dls)	Nano-ZS90	Malvern, UK
Transmission electron microscope (tem)	JEM-2100F	JEOL, Japan
Field emission scanning electron microscope (sem)	JSM-7800F Prime	JEOL, Japan
Magnetic resonance hydrogen spectrum	Avance NEO 400	Bruker, Germany
Ultraviolet spectrophotometer （uv-vis）	UV2550	Shimadzu, Japan
Enzyme-linked immunosorbent assay reader	SPECTROstar Nano	BMG LABTECH, Germany
Rotational rheometer	HAAKE RheoWin	Thermo Fisher Scientific, USA
Fourier transform infrared spectroscopy（ft-ir）	Nicolet 5700	Thermo Fisher Scientific, USA
fLuorescence microscope	Axio Observer	Carl Zeiss, Germany
Laser confocal microscope	SP8	Leica, Germany
fLow cytometer	NovoCyte	Agilent, USA
In vivo imaging device	IVIS Lumina LT	Ruifudi Biomedical (Shanghai) Co., Ltd., China
Pathological tissue drying oven	PHY-III	Changzhou Zhongwei

		Electronic Instrument Co., Ltd., China
Slide scanning imaging system	SQS-600P	Shenzhen Shengqiang Technology Co., Ltd., China

References
